# Net39 protects muscle nuclei from mechanical stress during the pathogenesis of Emery-Dreifuss muscular dystrophy

**DOI:** 10.1172/JCI163333

**Published:** 2023-07-03

**Authors:** Yichi Zhang, Andres Ramirez-Martinez, Kenian Chen, John R. McAnally, Chunyu Cai, Mateusz Z. Durbacz, Francesco Chemello, Zhaoning Wang, Lin Xu, Rhonda Bassel-Duby, Ning Liu, Eric N. Olson

**Affiliations:** 1 Department of Molecular Biology,; 2 Hamon Center for Regenerative Science and Medicine,; 3 Senator Paul D. Wellstone Muscular Dystrophy Cooperative Research Center,; 4 Quantitative Biomedical Research Center, Peter O’Donnell Jr. School of Public Health, and; 5 Department of Pathology, University of Texas (UT) Southwestern Medical Center, Dallas, Texas, USA.

**Keywords:** Muscle Biology, DNA repair, Gene therapy

## Abstract

Mutations in genes encoding nuclear envelope proteins lead to diseases known as nuclear envelopathies, characterized by skeletal muscle and heart abnormalities, such as Emery-Dreifuss muscular dystrophy (EDMD). The tissue-specific role of the nuclear envelope in the etiology of these diseases has not been extensively explored. We previously showed that global deletion of the muscle-specific nuclear envelope protein NET39 in mice leads to neonatal lethality due to skeletal muscle dysfunction. To study the potential role of the *Net39* gene in adulthood, we generated a muscle-specific conditional knockout (cKO) of *Net39* in mice. cKO mice recapitulated key skeletal muscle features of EDMD, including muscle wasting, impaired muscle contractility, abnormal myonuclear morphology, and DNA damage. The loss of *Net39* rendered myoblasts hypersensitive to mechanical stretch, resulting in stretch-induced DNA damage. *Net39* was downregulated in a mouse model of congenital myopathy, and restoration of *Net39* expression through AAV gene delivery extended life span and ameliorated muscle abnormalities. These findings establish NET39 as a direct contributor to the pathogenesis of EDMD that acts by protecting against mechanical stress and DNA damage.

## Introduction

The nuclear envelope plays critical roles in mechanotransduction and chromatin organization. In muscle fibers, nuclei are subjected to constant mechanical stress, and the envelope maintains nuclear architecture and integrity. The importance of a functional nuclear envelope in muscle is evidenced by the diverse human genetic disorders caused by mutations in genes encoding nuclear envelope proteins and the nuclear lamina. These diseases, such as Emery-Dreifuss muscular dystrophy (EDMD), are collectively known as nuclear envelopathies and laminopathies. EDMD is primarily caused by mutations in the genes LMNA and EMD, encoding lamin A/C and emerin, respectively (1). EDMD and other envelopathies are characterized by muscle abnormalities, including progressive skeletal muscle wasting and cardiac arrhythmias, even though most nuclear envelope proteins, including lamin A/C and emerin, are ubiquitously expressed.

Multiple mechanisms have been suggested for the pathogenesis of EDMD in muscle. The mechanotransduction model proposes that myonuclei are highly susceptible to damage because of the high mechanical strain in contractile tissues. Upon mechanical stretch, loss of nuclear envelope integrity can cause DNA damage and nuclear rupture (2). Alternatively, the gene expression model proposes that nuclear envelope proteins primarily regulate genome organization and gene expression, such that envelopathies cause pathological changes in transcription. Importantly, both pathways are interconnected, as mechanical stretch and DNA damage regulate gene expression (3, 4).

Another potential contributor to the phenotype of EDMD is the disruption of muscle-specific envelope proteins, which could render myonuclei particularly sensitive to pathogenic changes. Our previous study characterized the role of the muscle-specific nuclear envelope transmembrane protein 39 (NET39) in mice (5). *Net39*, also annotated as *Plpp7* or *Ppapdc3*, has been shown to regulate chromatin positioning and myoblast differentiation in vitro (6). We showed that *Net39* is essential for maintaining the integrity of the nuclear envelope and chromatin architecture in mice. *Net39*-KO mice were severely runted, died before weaning age, and presented with profound changes in nuclear morphology, chromatin accessibility, and gene expression. *NET39* is downregulated in human EDMD muscles and in primary mouse myoblasts with the *Lmna* ΔK32 mutation, which causes severe congenital myopathy (LMNA-related congenital muscular dystrophy) (7, 8). These findings implicate *Net39* in the muscle etiology of EDMD.

In this study, we sought to understand the role of *Net39* in adult skeletal muscle. We show that conditional knockout (cKO) of *Net39* in adult skeletal muscle causes a progressive, myopathic phenotype reminiscent of EDMD and restoration of *Net39* expression in *Lmna* ΔK32 mice through AAV-mediated gene delivery ameliorates the phenotype and extends life span. *Net39* maintains nuclear integrity, and its absence causes DNA damage exacerbated by mechanical stretch. By transcriptional analysis, we identified increased expression of DNA damage–related genes and myocyte-specific enhancer factor 2C (*Mef2c*) activation as key contributors to the myopathic phenotype. These findings provide insights into the role of *Net39* in EDMD pathogenesis through regulation of nuclear envelope integrity, genome stability, and Mef2c activity.

## Results

### Deletion of Net39 in adult muscle causes muscle wasting and weakness.

**Figure 1:**
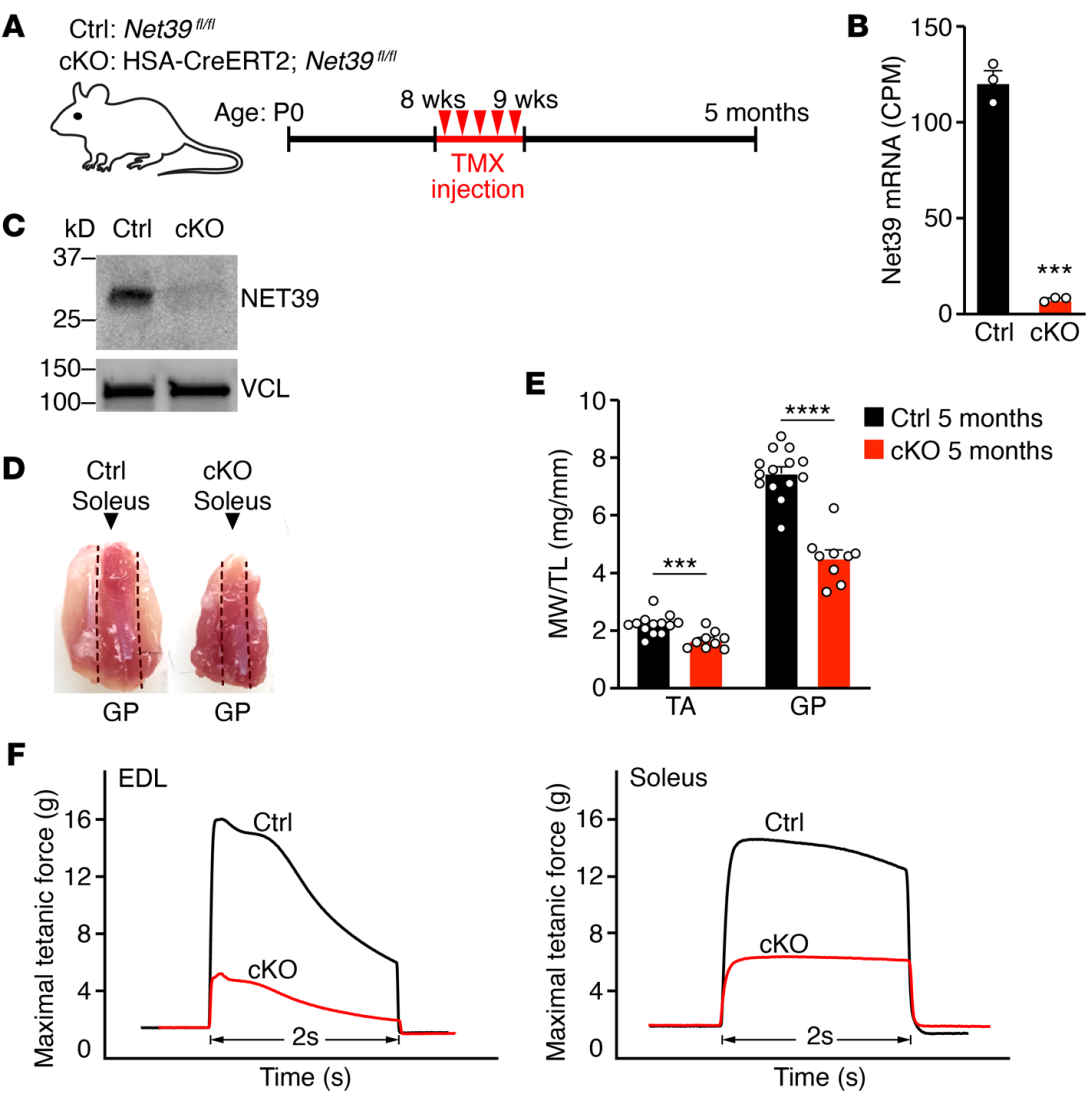
Conditional deletion of *Net39* in adult skeletal muscle leads to muscle wasting and weakness. (**A**) Experimental design for deletion of *Net39* in adult skeletal muscle. *Net39* cKO (cKO) and Ctrl mice were injected with tamoxifen for 5 consecutive days (red triangles) starting at 8 weeks of age and analyzed at 5 months of age. (**B**) *Net39* transcript abundance measured by bulk RNA-Seq of GP muscle at 5 months of age in cKO and Ctrl mice. CPM, counts per million. *n* = 3 mice. (**C**) Western blot analysis showing loss of NET39 protein in quadriceps muscle in cKO mice at 3 months of age. VCL protein was used as the loading control. (**D**) Gross morphology of Ctrl and cKO GP and soleus muscles at 5 months of age. Dotted lines mark the boundaries of the soleus. (**E**) Muscle weight (MW) to tibia length (TL) ratios for the indicated muscles in Ctrl and cKO mice at 5 months of age. *n* = 9–13 mice per group. (**F**) Ex vivo contraction assay to measure maximal tetanic force of the indicated muscles in Ctrl (black) and cKO (red) muscles at 5 months of age. Unpaired, 2-tailed Student’s *t* tests were performed for **B** and **E**. ***P < 0.001; ****P < 0.0001. Data are represented as mean ± SEM.

Global loss of *Net39* causes juvenile lethality, thus preventing studies on the role of *Net39* in adult mice (5). To understand the role of *Net39* in adult skeletal muscle, we generated a *Net39* conditional allele in mice (*Net39^fl^*) by inserting 2 loxP sequences flanking the first exon of *Net39* using CRISPR/Cas9-mediated homology-directed repair (Supplemental Figure 1A; supplemental material available online with this article; https://doi.org/10.1172/JCI163333DS1). *Net39^fl^* mice were bred with mice carrying a tamoxifen-inducible Cre recombinase transgene under control of the skeletal muscle–specific human skeletal actin promoter (HSA-CreERT2) (9). Tamoxifen was injected i.p. in *Net39^fl/fl^* (control group [Ctrl]) and HSA-CreERT2; *Net39^fl/fl^* mice (cKO) at 8 weeks of age for 5 consecutive days (Figure 1A). Genomic excision by Cre recombination was observed in skeletal muscle, but not in other tissues, by PCR analysis (Supplemental Figure 1B). Reduced *Net39* mRNA and protein levels in cKO muscles were confirmed by RNA-Seq (Figure 1B) and Western blot (Figure 1C). We observed no significant differences in muscle weight between cKO and Ctrl mice at 3 months of age (Supplemental Figure 1, C and D). However, by 5 months of age, male cKO mice displayed reduced mass of the gastrocnemius/plantaris (GP) and tibialis anterior (TA) muscles (Figure 1, D and E). The total body mass of cKO mice did not change at 5 (Supplemental Figure 1E) or 9 months of age (Supplemental Figure 1F). At 6 and 9 months, muscle weight of male cKO mice increased, but was still below that of Ctrl male mice (Supplemental Figure 1, G–I). There were no differences in survival between Ctrl and cKO mice. We also observed no differences in muscle weights of female cKO mice (Supplemental Figure 1J), indicating a sex bias of the phenotype. *Net39*-KO mice were previously shown to manifest a metabolic phenotype (5) that has been reported to be influenced by sex (10). Thus, all further experiments were performed on male mice at 5 months of age, unless indicated otherwise. Grip-strength tests showed that cKO mice generated lower muscle force (Supplemental Figure 1K), and ex vivo contractility assays revealed that soleus and extensor digitorum longus (EDL) muscles of cKO mice displayed reduced maximum tetanic force (Figure 1F), indicating impaired contractility.

**Figure 2:**
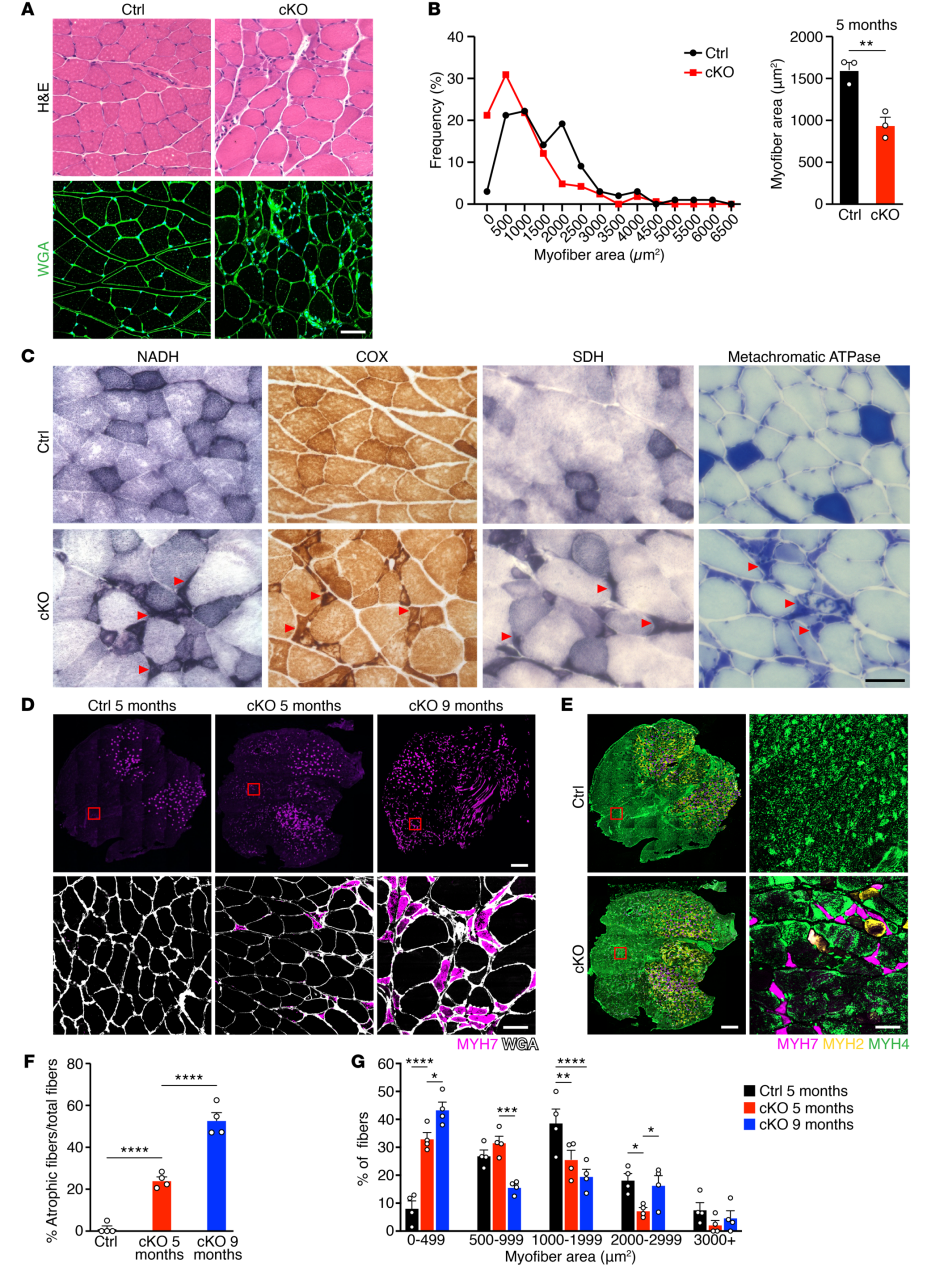
*Net39*-cKO muscles present small angular myofibers. (**A**) H&E (top) and WGA staining (bottom) of Ctrl and cKO GP muscles at 5 months of age. Scale bar: 50 μm. (**B**) Myofiber area distribution from WGA-stained GP sections (left) and average myofiber area (right) from Ctrl (black) and cKO (red) mice at 5 months of age. Unpaired, 2-tailed Student’s *t* test. *n* = 3 mice. (**C**) Immunohistochemistry of Ctrl and cKO GP muscles at 5 months of age for NADH, COX, SDH, and metachromatic ATPase staining. Type I fibers stain darkly, and type II fibers stain lightly. Red arrowheads indicate small angular myofibers in cKO muscle. Scale bar: 50 μm. (**D**) Immunostaining for MYH7 and WGA (bottom panels) of Ctrl and cKO GP muscles at 5 months of age and cKO GP muscle at 9 months of age. The magnified area on the bottom panels shows the presence of small angular fibers positive for MYH7 in cKO GP. The same area in Ctrl GP is usually devoid of MYH7-positive fibers. Scale bars: 500 μm (top); 50 μm (bottom). (**E**) Immunostaining for type I (MYH7), type IIa (MYH2), and type IIb (MYH4) myofibers of Ctrl and cKO GP muscles at 5 months of age. The magnified area on the right shows the presence of small angular fibers positive for MYH7 or MYH2 in cKO GP. Scale bars: 500 μm (left); 50 μm (right). (**F**) Quantification of small angular fibers over the total number of fibers from WGA staining in **D**. One-way ANOVA followed by Tukey’s multiple-comparisons test. *n* = 4 mice. (**G**) Quantification of myofiber areas from WGA staining in **D**. Two-way ANOVA followed by Tukey’s multiple-comparisons test. *P < 0.05; **P < 0.01; ***P < 0.001; ****P < 0.0001. *n* = 4 mice. Approximately 60-90 fibers per mouse were quantified for **B**, **F**, and **G**. Data are represented as mean ± SEM.

H&E and wheat germ agglutinin (WGA) staining of GP muscle revealed reduced myofiber cross-sectional area in cKO compared with Ctrl mice (Figure 2, A and B). In addition, cKO myofibers presented fiber-size disproportion, with small angular myofibers surrounded by larger ones (Figure 2A and Supplemental Figure 2A). The small angular myofibers stained strongly for nicotinamide adenine dinucleotide and hydrogen (NADH), cytochrome *c* oxidase (COX), succinate dehydrogenase (SDH), and metachromatic ATPase (Figure 2C). Immunohistochemistry of myosins revealed that the small angular myofibers mostly expressed type I myosin (MYH7) (Figure 2D and Supplemental Figure 2A), with a smaller number of fibers expressing type IIa myosin (MYH2) (Figure 2E). Immunofluorescence for the myopathic markers desmin, cleaved caspase-3, and embryonic myosin (MYH3) indicated that the small angular fibers were myopathic and potentially apoptotic (Supplemental Figure 2B). Ultrastructural analysis of GP muscles by electron microscopy revealed a subpopulation of myofibers with severely disorganized sarcomeres, which likely represent the small angular myofibers described above (Supplemental Figure 2C). Additional histological analysis at 5 and 9 months of age showed a progressive increase in small angular myofibers and centralized nuclei over time (Figure 2, F and G, and Supplemental Figure 2D) and an increase in small and large fibers in cKO muscle at 9 months of age, suggestive of compensatory hypertrophy (Figure 2G). cKO muscles were also more fibrotic, as evidenced by Masson’s trichrome staining (Supplemental Figure 2E). The small angular fibers were positive for CD11b, a marker of immune cell infiltration (11) (Supplemental Figure 2F). We observed no changes in circulating serum creatine kinase, indicating preserved plasma membrane integrity (Supplemental Figure 2G). Overall, these data indicate that adult loss of *Net39* leads to muscle wasting and a progressive myopathic phenotype that preferentially affects type I myofibers.

### Net39 deletion in adult muscle leads to impaired nuclear envelope integrity and DNA damage.

**Figure 3:**
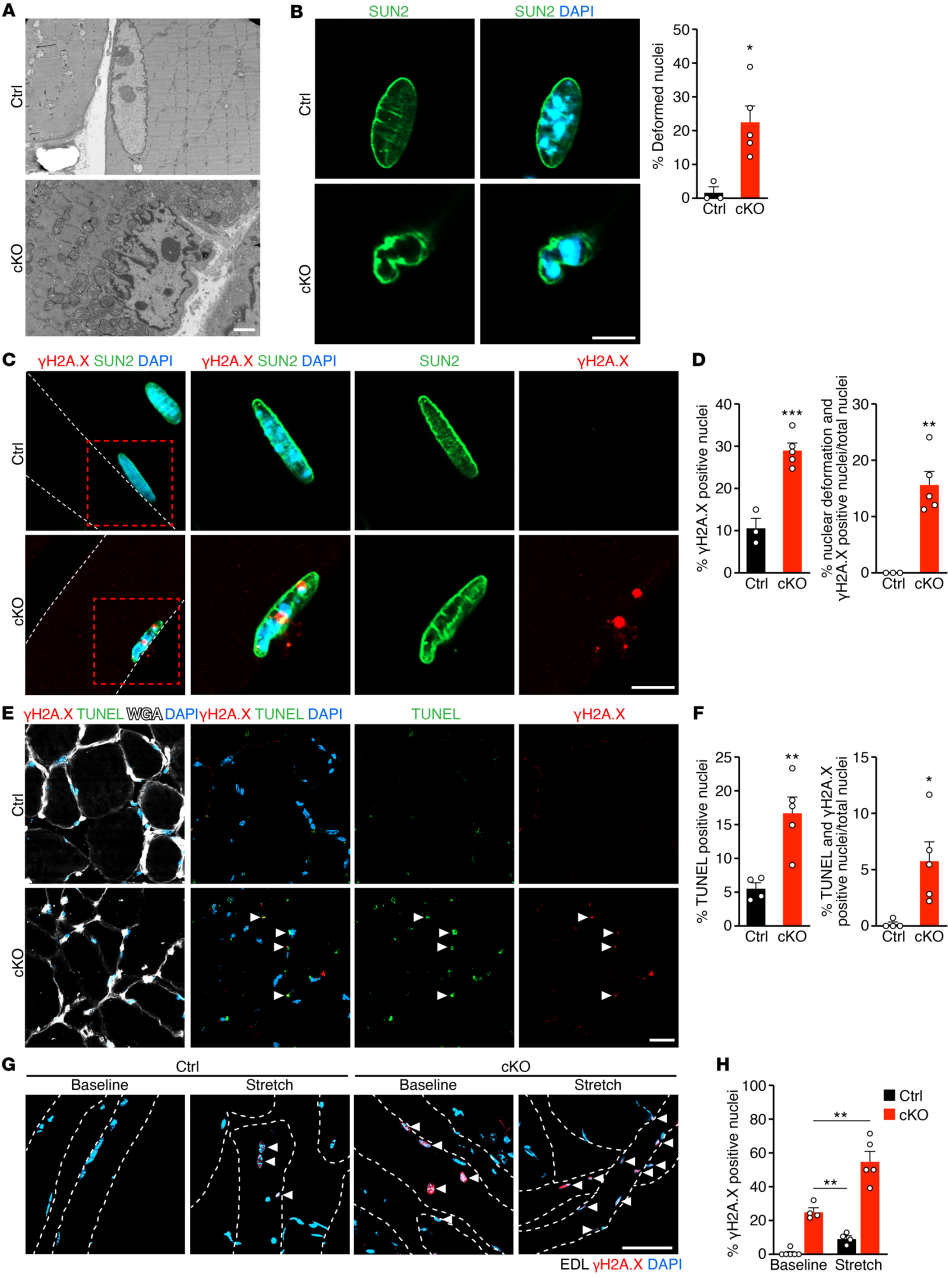
Loss of Net39 leads to impaired nuclear envelope integrity and DNA damage. (**A**) Electron micrographs showing a Ctrl nucleus and a cKO nucleus in GP muscles at 5 months of age. (**B**) Immunostaining of SUN2 (green) and DAPI (blue) in Ctrl and cKO GP muscles at 5 months of age (left). Quantification of deformed nuclei. *n* = 3–4 mice. (**C**) Immunostaining of SUN2 (green), γH2A.X (red), and DAPI (blue) in Ctrl and cKO GP muscles at 5 months of age. Dashed lines outline myofiber membranes. Magnified square area highlights a Ctrl and a cKO nucleus. (**D**) Quantification of γH2A.X-positive nuclei (left) and the percentage of deformed and γH2A.X-positive nuclei among the total number of nuclei (right) in Ctrl and cKO GP muscles. *n* = 3–5 mice. (**E**) TUNEL staining (green), γH2A.X (red), WGA (white), and DAPI (blue) immunostaining in Ctrl and cKO GP muscles at 5 months of age. Arrowheads indicate TUNEL- and γH2A.X-positive nuclei. (**F**) Quantification of TUNEL-positive nuclei (left) and the percentage of TUNEL- and γH2A.X-positive nuclei over the total number of nuclei (right) in **E**. *n* = 4–5 mice and approximately 200–800 nuclei per mouse. (**G**) γH2A.X staining (red) of isolated Ctrl and cKO EDL muscles at 5 months of age at baseline and after 30 minutes of ex vivo stretching. Dashed lines outline the myofiber membranes. Arrowheads indicate γH2A.X-positive nuclei. (**H**) Quantification of γH2A.X-positive nuclei over the total number of nuclei in **G**. *n* = 4–5 mice. Scale bars: 2 μm (**A**); 10 μm (**B** and **C**); 50 μm (**E** and **G**). *P < 0.05; **P < 0.01; ***P < 0.001. Data are represented as mean ± SEM. Unpaired, 2-tailed Student’s *t* test was performed for **B**, **D**, **F**, and **H**. 100 nuclei per mouse were analyzed for **B**, **D**, and **H**.

Electron microscopy of cKO muscles revealed that cKO myonuclei had jagged and deformed nuclear envelopes whereas no Ctrl nuclei displayed this phenotype (Figure 3A). Immunostaining for the nuclear envelope protein SUN2 showed that over 20% of nuclei in cKO myofibers were deformed (Figure 3B). The nuclear envelope deformations in cKO nuclei were associated with increased expression of phosphorylated H2A.X (γH2A.X), a marker for DNA damage (12). While nearly 30% of cKO nuclei were γH2A.X positive, approximately 16% of nuclei were both deformed and positive for γH2A.X (Figure 3, C and D). Western blot analysis showed increased γH2A.X protein levels in cKO GP muscles (Supplemental Figure 2, H and I). Activated, phosphorylated ATM (p-ATM), which phosphorylates γH2A.X (13), was also increased in cKO muscle (Supplemental Figure 2, H and I). TUNEL staining showed that approximately 16% of cKO nuclei were TUNEL positive and 6% of nuclei were positive for both γH2A.X and TUNEL, indicative of DNA damage and early apoptosis (Figure 3, E and F). Compromised nuclear integrity can cause nuclear envelope deformations and DNA damage (2, 14, 15). To determine whether cKO myonuclei were more fragile, myofibers from Ctrl and cKO EDL muscles were isolated and stretched ex vivo for 30 minutes; DNA damage was assessed by γH2A.X staining (Figure 3, G and H). cKO muscle fibers showed increased DNA damage at baseline, which was further exacerbated upon mechanical stretch. These results show that adult loss of *Net39* in skeletal muscle leads to compromised nuclear envelope integrity, increased DNA damage, and death of myonuclei.

### Protecting nuclei from mechanical stress prevents DNA damage in Net39-knockdown cells.

**Figure 4:**
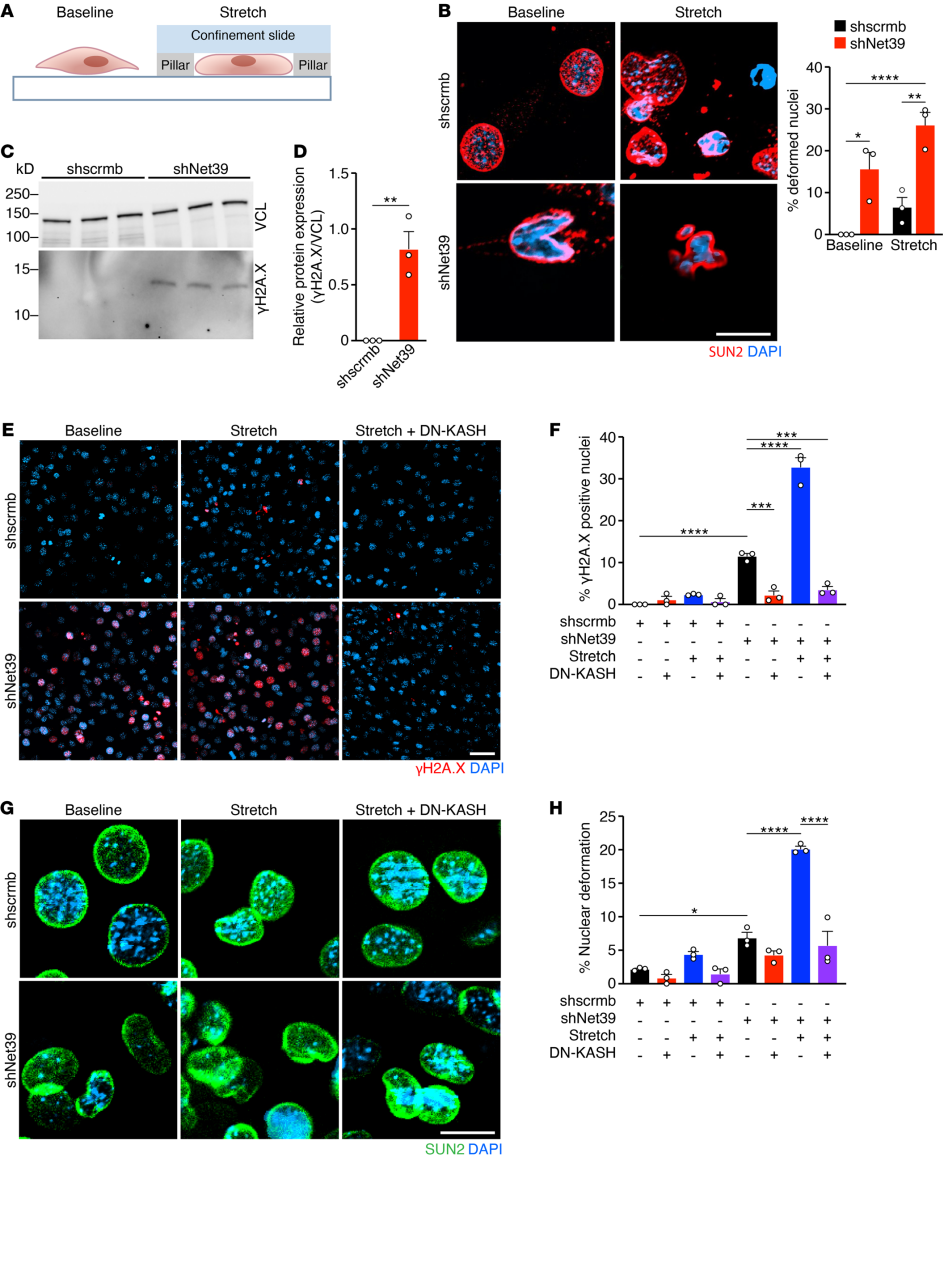
*Net39* knockdown in myoblasts causes DNA damage upon mechanical stress. (**A**) Schematic of cell compression system used to induce mechanical stretch of C2C12 myoblasts. Cells are confined within pillars on glass confinement slides. (**B**) Immunostaining of SUN2 (red) and DAPI (blue) in C2C12 myoblasts transduced with scrambled shRNA (shscrmb) and Net39 shRNA (shNet39) at baseline and after 1 hour of stretch. Quantification of deformed nuclei at baseline and after 1 hour of stretch. Approximately 100 cells per experiment. (**C**) Protein levels of γH2A.X normalized to VCL loading controls in shscrmb and shNet39 myoblasts as detected by Western blot analysis. (**D**) Densitometry analysis of the Western blots shown in **C**. (**E**) Immunostaining of γH2A.X (red) and DAPI (blue) in shscrmb and shNet39 C2C12 myoblasts at baseline, after 1 hour of stretch, and after 1 hour of stretch plus expression of DN-KASH, which disrupts the LINC complex. (**F**) Quantification of γH2A.X-positive nuclei in cell-stretching experiments with shscrmb and shNet39 C2C12 myoblasts and expression of DN-KASH. Approximately 100–300 cells per experiment. (**G**) Immunostaining of SUN2 (green) and DAPI (blue) in shscrmb and shNet39 C2C12 myoblasts at baseline, after 1 hour of stretch, and after 1 hour of stretch plus expression of DN-KASH, which disrupts the LINC complex. (**H**) Quantification of deformed nuclei in cell-stretching experiments with shscrmb and shNet39 C2C12 myoblasts and expression of DN-KASH. Deformed nuclei were identified using SUN2 staining from **G**. Approximately 100–300 cells per experiment. Scale bars: 10 μm (**B** and **G**); 50 μm (**E**). *P < 0.05; **P < 0.01; ***P < 0.001; ****P < 0.0001. Data are represented as mean ± SEM. Unpaired, 2-tailed Student’s *t* test was performed for **B** and **D**. One-way ANOVA followed by Tukey’s multiple-comparisons test was performed for **F** and **H**. *n* = 3 independent experiments were performed for **B**, **D**, **F**, and **H**.

To understand the contribution of *Net39* to the physical integrity of the nuclear envelope, we performed mechanical stretching experiments in C2C12 murine myoblasts. C2C12 cells were transduced with a shRNA to knock down *Net39* (shNet39) or a scrambled control (shscrmb) (Supplemental Figure 3A). Cells were then seeded in a confinement device, which applies stretch (16, 17) (Figure 4A), and nuclear deformations were quantified. The nuclear envelope integrity of shscrmb myoblasts was affected minimally by stretching for 1 hour (Figure 4B). In contrast, shNet39 myoblasts showed compromised nuclear envelope integrity at baseline, and stretch enhanced this phenotype, as evidenced by increased nuclear envelope deformations (Figure 4B). We also observed the appearance of γH2A.X-positive micronuclei in shNet39 myoblasts that increased following stretch (Supplemental Figure 3B), which is a sign of nuclear rupture (2). At baseline, shNet39 myoblasts already presented increased γH2A.X protein levels by Western blot analysis (Figure 4, C and D). Expression of DNA damage–induced genes such as *Trp53* and *Trp63* was elevated in shNet39 compared with shscrmb myoblasts at baseline (Supplemental Figure 3C). In addition, *Fas* and *Atf3* expression was increased after cell stretching in shNet39 myoblasts (Supplemental Figure 3C).

To further validate that *Net39* protects the nuclear envelope from physical stress, we uncoupled the nuclear envelope from cytosolic mechanical forces by overexpressing a dominant-negative KASH construct (DN-KASH). DN-KASH is a truncation of the protein nesprin-1 that disrupts the linker of nucleoskeleton and cytoskeleton (LINC) complex (18). Overexpression of DN-KASH reduced γH2A.X-positive nuclei to levels comparable to those of shscrmb (Figure 4, E and F) and rescued the percentage of deformed nuclei (Figure 4, G and H). Similar results were obtained with the microtubule stabilizer paclitaxel (19, 20) (Supplemental Figure 3, D–G). These data indicate that the high levels of DNA damage after *Net39* knockdown are primarily caused by increased sensitivity to mechanical stretch and that protecting nuclei from mechanical stress rescues the defects caused by loss of *Net39* in vitro.

### Loss of Net39 activates a pathological gene expression program in myonuclei.

**Figure 5:**
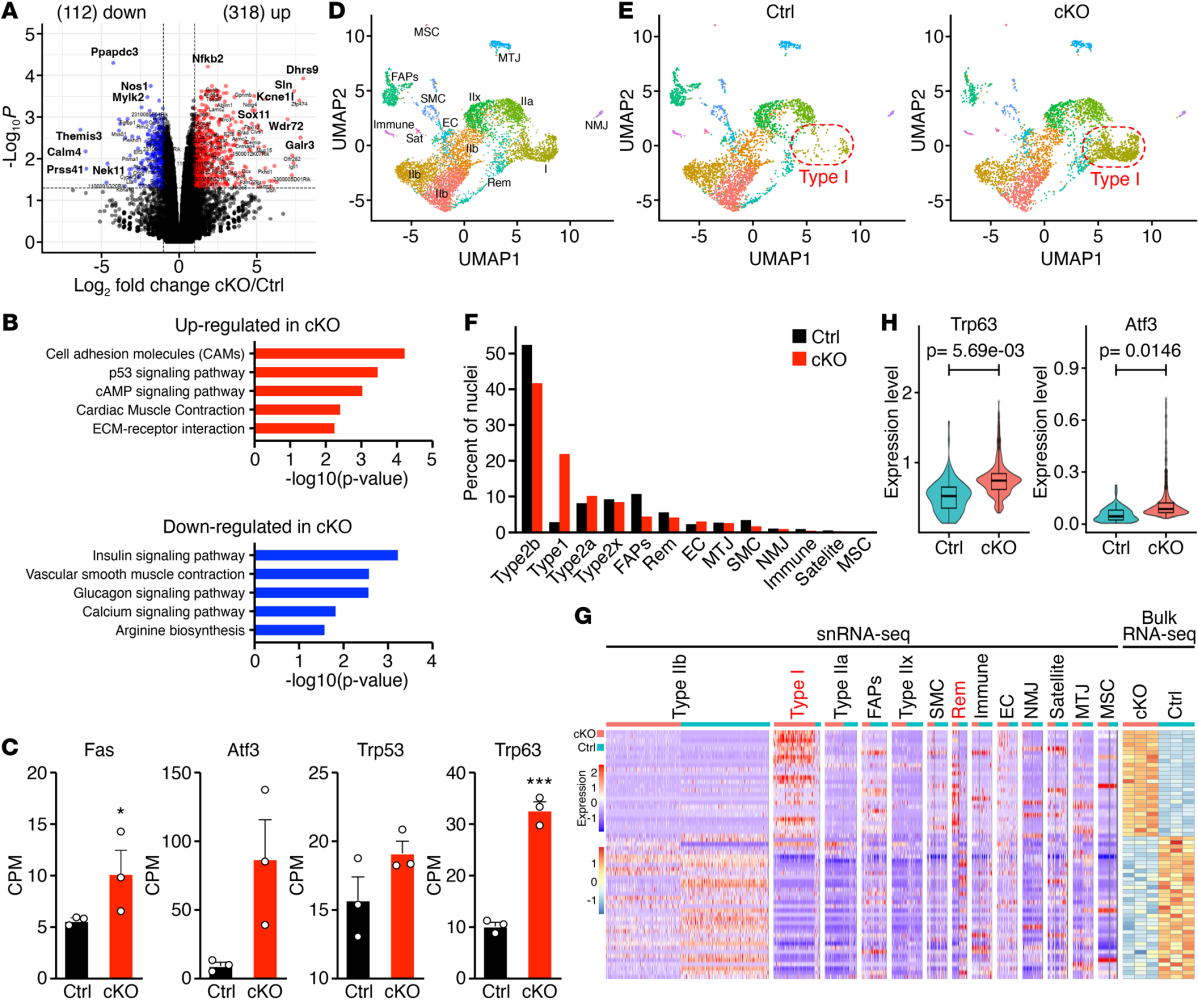
Transcriptomic analysis of cKO muscles reveals a pathologic myonuclear population. (**A**) Volcano plot illustrating the up- and downregulated genes in cKO GP compared with Ctrl GP muscles at 5 months of age by bulk RNA-Seq. A cut-off of fold change greater than 2 and an adjusted *P* value of less than 0.05 was set for the identification of differentially expressed genes. (**B**) Gene Ontology (GO) Pathway analysis of the up- (red) and downregulated (blue) genes in cKO muscle relative to Ctrl at 5 months of age by bulk RNA-Seq. (**C**) mRNA expression of DNA damage–induced genes in Ctrl and cKO GP muscle at 5 months of age detected by RNA-Seq. *P < 0.05; ***P < 0.001. Unpaired, 2-tailed Student’s *t* test. *n* = 3 mice. Data are represented as mean ± SEM. (**D**) UMAP visualization of nuclear transcriptomes from Ctrl and cKO GP muscles at 5 months of age by snRNA-Seq (7,296 nuclei) colored by cluster identity. SMC, smooth muscle cells; rem, remodeling myonuclei; EC, endothelial cells; NMJ, neuromuscular junction; MTJ, myotendinous junction; MSC, mesenchymal stem cells; Sat, satellite cells; IIx, type IIx myonuclei; IIa, type IIa myonuclei; IIb, type IIb myonuclei; I, type I myonuclei; immune, immune cells. (**E**) UMAP visualization of Ctrl (3,566 nuclei) (left) and cKO (3,730 nuclei) (right) nuclear transcriptomes by snRNA-Seq. Type I myonuclei are enclosed in red dashed lines. (**F**) Distribution plot showing the percentage of nuclei corresponding to the indicated populations in Ctrl (black) and cKO (red) samples. (**G**) Heatmaps showing the expression of the top 30 up- and downregulated genes by bulk RNA-Seq (right) and their expression in different nuclear populations by snRNA-Seq (left) in Ctrl and cKO GP muscles at 5 months of age. Color indicates *z* score. (**H**) Violin plots showing the expression of *Trp63* and *Atf3* in Ctrl (cyan) and cKO (pink) type I myonuclei.

To investigate the consequences of the loss of nuclear integrity in cKO nuclei, we profiled transcriptional changes caused by adult loss of *Net39* in skeletal muscle. Transcriptomic analysis was performed by bulk RNA-Seq in GP muscles. We identified 318 upregulated genes and 112 downregulated genes in cKO muscle (Figure 5A). Pathway analysis of bulk RNA-Seq revealed that p53 signaling was among the most upregulated pathways in cKO muscle (Figure 5B). This pathway includes DNA damage–induced genes, such as *Fas*, *Atf3,* *Trp53*, and *Trp63* (21), (Figure 5C).

To understand the heterogeneous changes in cKO muscle, single-nucleus RNA-Seq (snRNA-Seq) was performed on nuclei extracted from Ctrl and cKO GP muscles (Supplemental Figure 4A). Unsupervised clustering identified distinct nuclear populations that were assigned to biological populations based on the expression of marker genes (Figure 5D). The relative abundance of each cluster within Ctrl and cKO samples was then analyzed (Figure 5, E and F). cKO muscles showed a pronounced increase in type I myonuclei. Other populations declined, including type IIb myonuclei and fibro-adipogenic progenitors (FAPs) (Figure 5F). We identified a myonuclear population previously proposed to be involved in muscle fiber repair and remodeling (22), characterized by high expression of *Trp63* and the E3 ubiquitin ligases *Fbxo32/Atrogin-1* and *Trim63/MuRF1*. This cluster does not express a single myosin isoform, but instead can occasionally express *Myh1* and *Myh2* in the same nuclei (Supplemental Figure 4B).

Next, we integrated bulk RNA-Seq and snRNA-Seq data sets and observed that the most significant changes in gene expression from bulk RNA-Seq could be attributed to transcriptional changes in type I myonuclei (Figure 5G). Differential gene expression (DEG) and pathway analyses of the type I population showed that cKO type I myonuclei underwent a general dysregulation of muscle structural genes (e.g., *Myh7*, *Mylk4*) compared with Ctrl type I myonuclei (Supplemental Figure 4, C and D). These myonuclei also expressed greater levels of DNA damage–induced genes (*Atf3*, *Trp63)* (Figure 5H and Supplemental Figure 4E), and pathway analysis performed on the upregulated genes revealed that p53 regulated genes were induced (Supplemental Figure 4D). These findings suggest that as cKO myonuclei progressively accumulate stretch-induced DNA damage, they transition into a pathological myonuclear population transcriptionally similar to type I myonuclei.

Importantly, we did not observe increased expression of these DNA damage–related genes in mouse models of other muscle disorders (Supplemental Figure 4, F and G), including Duchenne muscular dystrophy (*Dmd* ΔEx51 mice) and nemaline myopathy (*Klhl41*-KO mice) (23, 24). Therefore, DNA damage likely contributes to the pathogenesis of EDMD, but not other myopathies in which nuclear envelope integrity is not the main driver of the disease.

### Mef2c activation underlies dysregulated gene expression in cKO muscle.

**Figure 6:**
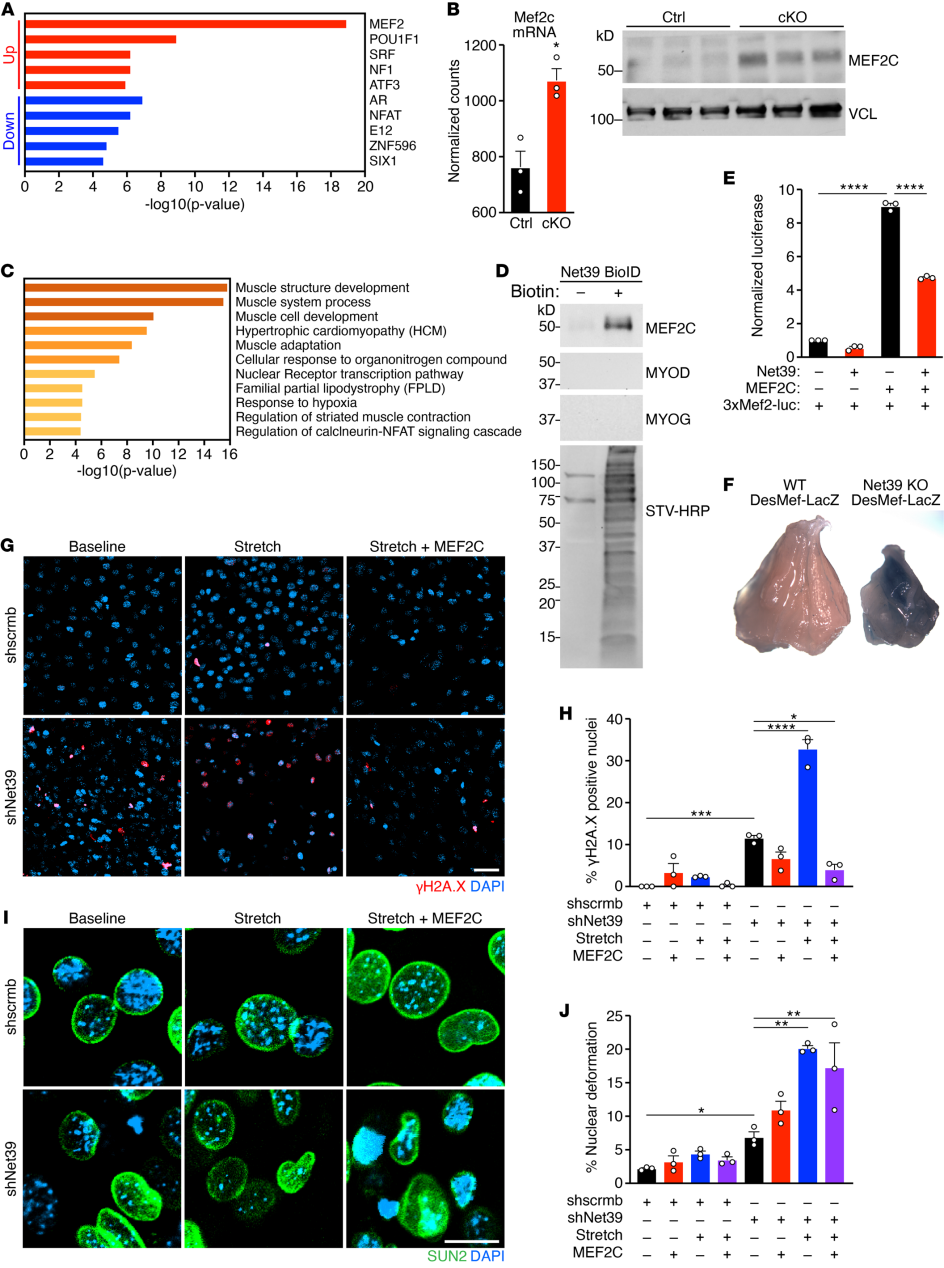
*Net39* regulates *Mef2c* activity to protect against DNA damage induced by mechanical stress. (**A**) Upstream regulator analysis of transcription factors (TFs) for the upregulated (red) and downregulated (blue) genes from cKO myonuclei identified by snRNA-Seq. (**B**) Transcript levels of *Mef2c* mRNA detected by bulk RNA-Seq and protein levels of MEF2C and VCL loading control detected by Western blotting were measured in GP muscles from Ctrl and cKO mice. Unpaired, 2-tailed Student’s *t* test. *n* = 3 mice. (**C**) Top GO pathways enriched in *Mef2c* target genes that are upregulated in cKO myonuclei from snRNA-Seq. (**D**) Net39 BioID in C2C12 myotubes detects enrichment of biotinylated MEF2C, but not the myogenic transcription factors MYOD and MYOGENIN (MYOG). Total biotinylated proteins were detected using streptavidin-HRP (STV-HRP). (**E**) Luciferase activity was measured in the presence (+) or absence (–) of MEF2C and NET39 and was normalized to X-gal. (**F**) Whole-mount X-gal staining of GP muscles at P17 performed in WT and *Net39*-KO mice expressing the DesMef-LacZ transgene. (**G**) Immunostaining of γH2A.X (red) and DAPI (blue) in shscrmb and shNet39 C2C12 myoblasts at baseline, after 1 hour of stretch, and after 1 hour of stretch plus overexpression of MEF2C. Experiment and analysis in Figure 4, E–H, were performed contemporaneously with **G**–**J**. (**H**) Quantification of γH2A.X-positive nuclei in **G**. (**I**) Immunostaining of SUN2 (green) and DAPI (blue) in shscrmb and shNet39 C2C12 myoblasts at baseline, after 1 hour of stretch, and after 1 hour of stretch plus overexpression of MEF2C. (**J**) Quantification of deformed nuclei using SUN2 staining from **G**. Scale bars: 50 μm (**G**); 10 μm (**I**). *P < 0.05; **P < 0.01; ***P < 0.001; ****P < 0.0001. Data are represented as mean ± SEM. One-way ANOVA followed by Tukey’s multiple-comparisons test and 3 independent experiments were performed for **E**, **H**, and **J**. n = 3 independent experiments were performed for **E**, **H**, and **J**.

We sought to understand the molecular mechanism underpinning the pathological gene expression program in cKO myonuclei. Nuclear envelope proteins can regulate genomic regions known as lamin-associated domains (LADs) (25, 26). LADs have been proposed to regulate gene expression, but this concept remains controversial (25, 27–29). We performed ChIP-Seq on C2C12 myotubes expressing Ty1-tagged NET39 and found that NET39 bound to the genome overlaid with LADs (Supplemental Figure 5, A and B). We then determined whether loss of NET39 caused changes in LADs that would correlate with gene expression or chromatin accessibility. For this purpose, we analyzed previously published data sets on *Net39*-KO mice (5). However, we did not observe any correlation between genes within LADs and their changes in gene expression or chromatin accessibility in *Net39*-KO mice (data not shown). To unbiasedly interrogate the molecular pathways altered in cKO muscle, we merged the transcriptomic changes of all myonuclei populations from snRNA-Seq data and performed upstream regulator analysis to identify transcription factor binding. This analysis showed that the upregulated genes in cKO myonuclei were Mef2 target genes (Figure 6A). Furthermore, bulk RNA-Seq and Western blot analysis revealed increased *Mef2c* transcript and protein levels in cKO muscles (Figure 6B). snRNA-Seq showed that *Mef2c* expression was most significantly enriched in type I myonuclei (Supplemental Figure 6A). Among Mef2 paralogs, the upregulation was most evident for *Mef2c* (Supplemental Figure 6B). Pathway analysis of the upregulated *Mef2c* target genes showed that they control muscle development, consistent with the known function of *Mef2c* (Figure 6C and Supplemental Figure 6C). These results indicate that following the loss of *Net39*, *Mef2c* is induced and plays a significant role in regulating the disease-associated changes in gene expression.

It has been shown that a pool of MEF2C interacts with the nuclear pore complex to regulate myoblast differentiation (30). Proximity labeling BioID experiments using a NET39-mTurbo fusion protein in C2C12 myotubes revealed that a fraction of the total MEF2C protein was in close proximity to NET39 (Figure 6D), but not other myogenic factors such as MYOD or MYOG. To assess the regulation of *Mef2c* activity by *Net39* in vitro, we used a luciferase reporter under control of 3 MEF2C-binding sites from the mouse *Des* enhancer (31). HEK293T cells were used for luciferase assays, to avoid interference with endogenous muscle-specific regulators of gene expression. Expression of *Mef2c* induced luciferase reporter activity, and coexpression of *Net39* was sufficient to inhibit the reporter (Figure 6E). For mouse experiments, we used DesMef-LacZ mice, which carry a LacZ reporter expression cassette under control of the same *Des* enhancer. When DesMef-LacZ mice were bred to *Net39*-KO mice, we observed increased X-gal staining in *Net39*-KO GP muscles compared with WT (Figure 6F). Prior studies showed that *Mef2c* activity is silenced in normal adult skeletal muscle and forced *Mef2c* activation is sufficient to cause fiber-type switching in vivo (32). Therefore, the regulation of *Mef2c* activity by *Net39* could contribute to the activation of the abnormal type I fiber gene program observed in cKO mice. We did not observe increased *Mef2c* expression in mouse models for Duchenne muscular dystrophy and nemaline myopathy (23, 24) (Supplemental Figure 6, D and E). Therefore, *Mef2c* activation is likely specific for certain myopathies, such as EDMD.

*Mef2c* has been reported to induce the expression of DNA repair genes in lymphoid cells to reduce DNA damage (33). We hypothesized that *Mef2c* activation could be a protective response to increased DNA damage. To test this, in vitro cell-stretching experiments were performed with shNet39 and shscrmb C2C12 cells overexpressing *Mef2c* (Figure 6G). *Mef2c* overexpression prevented stretch-induced DNA damage in both shscrmb and shNet39 C2C12 cells (Figure 6, G and H), but did not reduce nuclear envelope deformations (Figure 6, I and J). We also observed that *Mef2c* expression increased following cell stretch of C2C12 myoblasts (Supplemental Figure 6F) and paclitaxel treatment (Supplemental Figure 3H). Together, these results suggest that *Mef2c* expression can be induced by mechanical stress as a mechanism to alleviate DNA damage and that a pool of MEF2C may be directly regulated by the nuclear envelope and become active in response to stretch.

### NET39 downregulation, DNA damage, and MEF2C induction in human EDMD.

**Figure 7:**
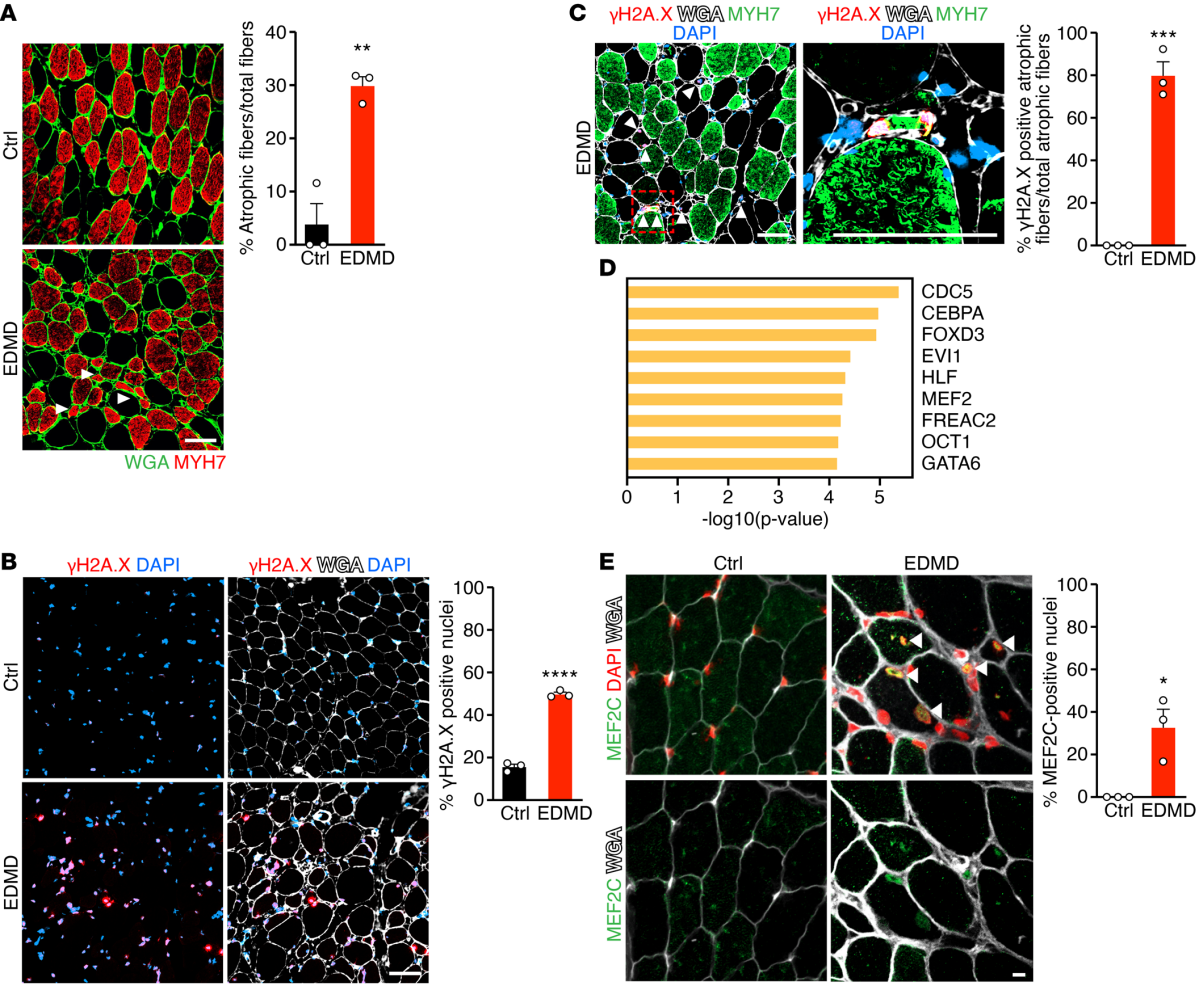
DNA damage and MEF2C induction in human EDMD. (**A**) Immunostaining for MYH7 (red) and WGA (green) in healthy Ctrl and EDMD patient muscle biopsies (left). White arrowheads indicate atrophic myofibers. Scale bar: 50 μm. Quantification of percentage of atrophic myofibers out of the total number of myofibers (right). **P < 0.01. Approximately 60–100 myofibers per sample. (**B**) Immunostaining for γH2A.X (red), WGA (white), and DAPI (blue) in Ctrl and EDMD muscle biopsies. Scale bar: 50 μm (left). Quantification of γH2A.X-positive nuclei (right). ****P < 0.0001. Approximately 50–100 nuclei analyzed per sample. (**C**) Immunostaining for γH2A.X (red), MYH7 (green), WGA (white), and DAPI (blue) in EDMD muscle biopsies (left). γH2A.X is mostly detected in small angular myofibers that are also MYH7 positive. White arrowheads indicate γH2A.X-positive nuclei. An atrophic myofiber with γH2A.X-positive nuclei (red box) is enlarged (middle). Quantification of γH2A.X-positive fibers that are also atrophic among all atrophic myofibers in Ctrl and EDMD muscle (right). ***P < 0.001. Scale bars: 50 μm. Approximately 60–100 myofibers analyzed per sample. (**D**) Upstream regulator analysis of TFs for the most upregulated genes from a published human EDMD patient microarray (34, 35). (**E**) Immunostaining of MEF2C (green), WGA (white), and DAPI (red) in Ctrl and EDMD muscle biopsies. White arrowheads indicate MEF2C-positive nuclei in EDMD. Scale bar: 10 μm. Quantification of MEF2C-positive nuclei (right). *P < 0.05. Approximately 50–100 nuclei analyzed per sample. Unpaired, 2-tailed Student’s *t* test was performed for **A**, **B**, **C**, and **E**. *n* = 3 human samples were analyzed for **A**, **B**, **C**, and **E**. Data are represented as mean ± SEM.

We previously reported that *NET39* is downregulated in human EDMD muscles from patients harboring different *LMNA* mutations (mutations described in Methods) (5). Additional analysis of EDMD muscle biopsies where *NET39* was downregulated revealed similarities with cKO mice. Histologically, EDMD muscle biopsies showed fiber size disproportion, with small angular myofibers positive for MYH7, similar to the ones in cKO muscles (Figure 7A). γH2A.X staining also showed a significant increase in DNA damage in EDMD myonuclei compared with Ctrl (Figure 7B). Increased γH2A.X staining was predominant in small angular myofibers, with approximately 80% of these fibers being positive for γH2A.X (Figure 7C). Upstream regulator analysis of upregulated genes from human EDMD patient microarray data sets (34, 35) revealed that MEF2 was among the main transcriptional regulators (Figure 7D). Immunofluorescence on EDMD patient biopsies showed increased MEF2C-positive nuclei compared with healthy Ctrls (Figure 7E), resembling the MEF2C activation seen in cKO muscles. These results highlight the histopathological and molecular similarities between human EDMD and cKO mice, in which *Net39* was deleted in adult skeletal muscle.

### AAV9-Net39 gene therapy ameliorates the phenotype of Lmna ΔK32 mice.

Currently, there is no cure for EDMD. We showed previously that *NET39* gene expression is decreased in EDMD patients (5), raising the possibility of restoring *NET39* expression to improve muscle defects in EDMD. We first determined whether *Net39* gene therapy could rescue the severe phenotype of *Net39*-KO mice. *Net39* was cloned into an adeno-associated virus (AAV) backbone under control of the muscle-specific muscle creatine kinase promoter (CK8e) (36) and packaged into AAV9, a viral vector with tropism for striated muscle (37). We first delivered AAV-Net39 or AAV-TdTomato (AAV-TdTo) control virus i.p. into *Net39*-KO mice at P6 (Supplemental Figure 7A). Quantitative reverse-transcription PCR (qRT-PCR) analysis showed a 45-fold increase in *Net39* transcript levels in *Net39*-KO mice injected with AAV-Net39 compared with WT (Supplemental Figure 7B). Importantly, 83% (5 out of 6) of *Net39*-KO mice injected with AAV-Net39 survived to adulthood past 3 months of age (Supplemental Figure 7, C and D). However, mice still presented lower muscle weight than WT Ctrls (Supplemental Figure 7E). Histologically, *Net39*-KO mice treated with AAV-Net39 showed a significant reduction in centralized nuclei (Supplemental Figure 7F), absence of fibrosis (Supplemental Figure 7G), and decreased NADH oxidative staining (Supplemental Figure 7H). Overall, these data indicate that AAV-Net39 treatment is sufficient to rescue the early lethality and severe muscle phenotype caused by loss of *Net39* expression.

**Figure 8:**
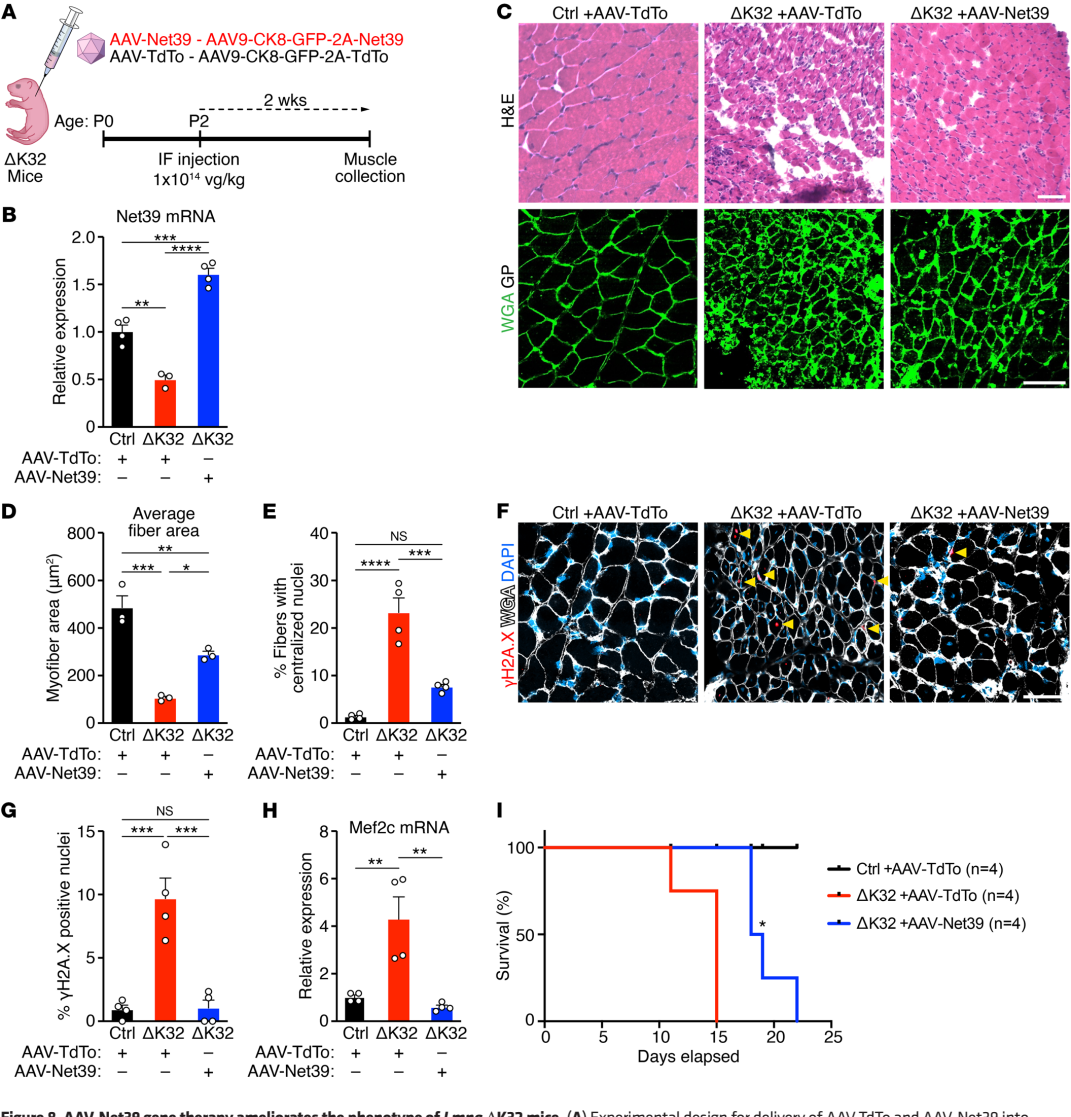
AAV-Net39 gene therapy ameliorates the phenotype of *Lmna* ΔK32 mice. (**A**) Experimental design for delivery of AAV-TdTo and AAV-Net39 into *Lmna* ΔK32 mice (ΔK32). 1 × 10^14^ viral genome (vg)/kg mouse weight of AAV was injected via facial vein (IF) at P2 and muscles were analyzed after 2 weeks. (**B**) *Net39* mRNA expression in GP muscles at P16 from Ctrl mice injected with AAV-TdTo (black) and ΔK32 mice injected with AAV-TdTo (red) or AAV-Net39 (blue). *n* = 3–4 mice per group. (**C**) H&E staining of GP muscles at P16 from Ctrl and ΔK32 mice injected with AAV-TdTo or AAV-Net39 (top). WGA staining of GP muscles from Ctrl and ΔK32 mice injected with AAV-TdTo or AAV-Net39 (bottom). (**D**) Quantification of average myofiber area from WGA-stained sections in **C**. *n* = 3 mice per group and 30–100 myofibers quantified per mouse. (**E**) Quantification of fibers with centralized nuclei from H&E-stained section in **C**. *n* = 4 mice per group and 200–400 nuclei quantified per mouse. (**F**) Immunostaining for γH2A.X (red), WGA (white), and DAPI (blue) in GP sections at P16 from Ctrl mice injected with AAV-TdTo and ΔK32 mice injected with AAV-TdTo or AAV-Net39. (**G**) Quantification of γH2A.X-positive nuclei from **F**. *n* = 4 mice per group and 100–200 nuclei quantified per mouse. (**H**) *Mef2c* mRNA expression in GP muscles at P16 from Ctrl mice injected with AAV-TdTo (black) and ΔK32 mice injected with AAV-TdTo (red) or AAV-Net39 (blue). *n* = 4 mice per group. (**I**) Survival curves of Ctrl mice injected with AAV-TdTo (black) and ΔK32 mice injected with AAV-TdTo (red) or AAV-Net39 (blue). log-rank (Mantel-Cox) test. *n* = 4 mice per group. Scale bars: 50 μm. *P < 0.05; **P < 0.01; ***P < 0.001; ****P < 0.0001. Data are represented as mean ± SEM. One-way ANOVA followed by Tukey’s multiple-comparisons test was performed for **B**, **D**, **E**, **G**, and **H**.

To study the significance of AAV-Net39 gene therapy in a broader context, we sought to restore *Net39* expression in a disease model where *Net39* is downregulated. We selected the *Lmna* ΔK32 (ΔK32) mouse model for initial experiments. Mice harboring the ΔK32 allele manifest severe laminopathy (LMNA-related congenital muscular dystrophy) that closely phenocopies the lethal phenotype of *Net39*-KO mice (7). Additionally, recent studies have shown that ΔK32 mouse myoblasts downregulate *Net39* expression (8). We performed facial vein injection of AAV-Net39 (or AAV-TdTo) in ΔK32 mice at P2 (Figure 8A). qRT-PCR analysis showed that *Net39* transcript levels were reduced by 50% in ΔK32 mice compared with Ctrl mice, and AAV-Net39 injection was sufficient to restore physiological *Net39* transcript levels in ΔK32 mice (Figure 8B). AAV-Net39–treated ΔK32 mice showed a mild increase in myofiber area (Figure 8, C and D), with reduced levels of centralized nuclei (Figure 8E). The percentage of γH2A.X-positive nuclei was also reduced in GP muscles of AAV-Net39–treated ΔK32 mice compared with AAV-TdTo–treated ΔK32 mice (Figure 8, F and G). The transcript levels of *Mef2c* were significantly increased in ΔK32 mice, and AAV-Net39 delivery restored *Mef2c* expression to Ctrl levels (Figure 8H). However, AAV-Net39 was not sufficient to completely rescue the phenotype, as ΔK32 mice treated with AAV-Net39 still died before weaning and did not fully recover body weight to appreciable levels (Supplemental Figure 8). Nevertheless, the life span of ΔK32 mice treated with AAV-Net39 was significantly increased compared with that of ΔK32 mice treated with AAV-TdTo virus (Figure 8I). Therefore, while the ΔK32 phenotype is molecularly complex, affects multiple organs, and initiates during embryonic development (7), postnatal delivery of AAV-Net39 was sufficient to improve the ΔK32 phenotype. In summary, postnatal restoration of *Net39* expression by AAV delivery ameliorated a model of severe laminopathy, directly implicating *Net39* in the pathogenesis of this disorder and potentially other diseases of the nuclear envelope in which *Net39* is downregulated.

## Discussion

Our data show that *Net39* is required to maintain the integrity of the nuclear envelope in adult muscles. Adult loss of *Net39* leads to nuclear deformations within myofibers, causing DNA damage, activation of a Mef2-dependent pathological gene expression program, muscle wasting, and impaired contraction. Loss of *Net39* affected nuclear envelope integrity and rendered myonuclei susceptible to mechanical stretch in vitro and in vivo, which was reflected in increased DNA damage. Thus, we conclude that *Net39* protects nuclei within muscle fibers against the mechanical stress caused by muscle contraction.

Envelopathies, such as EDMD, typically cause broad transcriptional changes in muscle tissues. It is not well understood which transcriptional changes contribute to the disease primarily and which are secondary. *NET39* is downregulated in human EDMD muscles, and we observed that cKO mice closely mimic the clinical manifestation of human EDMD. Importantly, we showed the direct contribution of *Net39* to disease progression in *Lmna* ΔK32 mice, an animal model of congenital myopathy in which *Net39* is downregulated (7, 8). Postnatal delivery of AAV-Net39 extended the life spans of *Lmna* ΔK32 mice and reverted part of the phenotype. However, there is a broad spectrum of mutations that cause EDMD and they differ in their phenotypes. For instance, *Net39* levels are not downregulated in *Lmna* H222P myoblasts, and the *Lmna* H222P mutation has a milder phenotype in mice and humans (8, 38). Our findings demonstrate that *Net39* is specifically involved in the pathogenesis of *Lmna* ΔK32 mice, but not necessarily other mutations. Furthermore, multiple *NET39* gene variants have been identified in patients with muscular dystrophies, but their significance remains unknown (39). Our findings suggest that mutations in *NET39* could also contribute to EDMD, either directly or as a modifier gene.

In the current study, we modeled mechanical stress by ex vivo myofiber stretching and in vitro compression of myoblasts using a cell-stretching device. We show that the loss of NET39 weakens the nuclear envelope, which becomes more prone to deformation and rupture following mechanical stimulation, as evidenced by induction of DNA damage and appearance of micronuclei (2). The positive correlation in cKO mice among nuclear deformations, increased γH2A.X, and increased TUNEL-positive nuclei indicates that genomic instability likely contributes to the deterioration and death of myofibers. Based on these data, we propose that *Net39* protects myonuclei and their respective myofibers against mechanical stress–induced damage. Uncoupling the nuclear envelope from external forces through DN-KASH expression or paclitaxel treatment reduced the DNA damage caused by *Net39* loss. DN-KASH gene therapy could potentially ameliorate the muscle phenotype in EDMD, and it has already shown promise in lamin-induced cardiomyopathy (40).

Transcriptional analysis of cKO and human EDMD samples showed the dysregulation of specific transcription factors and their target genes, particularly Mef2, which we validated in vitro and in vivo. Mef2 expression is induced by stretch, and DNA damage can regulate Mef2 activity (4, 33). Prior studies have reported that the nuclear envelope can regulate the activity of muscle-related transcription factors, such as *Mef2c* and *Myod1* (30, 41). MEF2C is recruited to the nuclear pore complex during myoblast differentiation to form transcriptionally active complexes (30), and we observed a potential interaction between NET39 and MEF2C by proximity labeling. Our evidence suggests that, besides an indirect compensatory mechanism, a pool of MEF2C likely interacts with NET39 and is recruited to the nuclear periphery, where it likely remains associated with heterochromatin and becomes repressed. Previous work from our laboratory demonstrated that expression of a hyperactive form of *Mef2c* in skeletal muscle promotes a fiber-type shift toward type I fibers (32). We identified a pathological type I myonuclear population with increased expression of DNA damage markers and dysregulation of Mef2-dependent muscle contractile genes as the primary contributor to the global changes in transcription. We did not observe an induction of *Mef2c* expression in other myopathy models, indicating that *Mef2c* activation is not a general myopathic response. Considering that *Mef2c* overexpression protected cells from DNA damage after depletion of *Net39*, we surmise that *Mef2c* activation may be an adaptive response to DNA damage. Together, these results suggest that loss of *Net39* can activate specific transcription factors in affected myonuclei, which account for the changes in transcription observed in EDMD and contribute to the phenotype.

Overall, our work demonstrates the essential role of *Net39* in protection of the nuclear envelopes within muscle fibers from the mechanical stress caused by contraction and highlights the contribution of muscle-specific nuclear envelope proteins to the pathogenesis of EDMD.

## Methods

### Conditional deletion of Net39 in adult mice.

Cas9 sgRNAs flanking the first exon of *Net39* were selected using the CRISPOR sgRNA design tool (42). sgRNAs were tested using an in vitro Cas9 cleavage assay (43). The following 2 sgRNAs were selected: no. 1: Net39-sgRNA-5′, TTTAATCCCAGGGACTGTAG; no. 2: Net39-sgRNA-3′, AGTAGTGTCTGTAACTGGTT.

The DNA templates used for homologous recombination and insertion of loxP sites were single-stranded oligodeoxynucleotide donors (ssODN) (Integrated DNA Technologies) with short asymmetric homology arms. Lowercase denotes genomic DNA sequence, and uppercase denotes loxP sites: no. 1: ssODN-Net39-loxp-5′, agaaggaggaggaggaaagaagaaaggggatagttgacttagccctagagacccaacatagagtgctctcagtgacatcaaaattcccctaGAATTCATAACTTCGTATAATGTATGCTATACGAAGTTATcagtccctgggattaaagtacccactgcccagccag; no. 2: ssODN-Net39-loxp-3′, caggggcagagtacaaagaggtcaaggggtgaatttgggggctctgctgagtggcctagtgtgatgtcttggtcaggttgttattcctaacGAATTCATAACTTCGTATAATGTATGCTATACGAAGTTATcagttacagacactactgaactgaccccaaattttc.

Zygotes were obtained from superovulating B6C3F1 female mice mated to B6C3F1 stud males. Cas9 mRNAs, Net39 sgRNAs, and ssODNs were injected into the pronucleus and cytoplasm of zygotes and transferred into pseudopregnant ICR female mice.

For genomic analysis, tail DNA was extracted from F0 mice and used to analyze the targeted regions by PCR. The allele containing a loxP site showed an increase of 40 bp in the length of the expected PCR product. The primers were designed as follows: primers no. 1 and no. 2 amplified the region containing the 5′ loxP site, and primers no. 3 and no. 4 amplified the region containing the 3′ loxP site. Primers no. 1 and no. 4 were used to amplify the region containing both loxP sites. Primers were as follows: no. 1: Net39-KI-5′-F, GGATAGTTGACTTAGCCCTAGAGACC; no. 2: Net39-KI-5′-R, AATAGAGCCCACCCTTACAGG; no. 3: Net39-KI-3′-F, CTGCTGCTAGGTGAGTGTGC; no. 4: Net39-KI-3′-R, GAACCTGGCAGTGTTGGTTT.

F0 mosaic mice were mated to C57BL/6N mice to generate mice heterozygous for the floxed *Net39* allele (*Net39^fl^*). *Net39^fl^* mice were crossed with mice harboring the HSA-CreERT2 transgene, which encodes a tamoxifen-inducible CreERT2 cassette specifically expressed in skeletal muscle, but not in satellite cells or the heart (9). The prior primers no. 1 and no. 2 were used for routine genotyping. The following primers were used to genotype the HSA-CreERT2 transgene: no. 1: HSA-CreERT2-F, AAGTTCGTTCACTCATGGA; no. 2: HSA-CreERT2-R, TCGACCAGTTTAGTTACCC.

Cre-mediated excision of the *Net39* locus was induced by tamoxifen (Sigma-Aldrich, T5648) reconstituted in 90% sesame oil and 10% ethanol at a concentration of 20 mg/mL. Eight-week-old male mice were injected with tamoxifen peritoneally at a dose of 100 mg/kg for 5 consecutive days. Cre-mediated deletion of *Net39* was verified in genomic DNA by GXL DNA Polymerase (Takara, R050B) long-range PCR using primers no. 1 and no. 4. The expected WT PCR product had a size of 2.3 kb, whereas the recombined allele was 905 bp. Serum was collected from heart puncture for creatine kinase measurements using VITROS Chemistry Products CK Slides on the VITROS 250 Chemistry System.

### Ex vivo electrophysiology.

EDL and soleus muscles were isolated from 5 month-old Ctrl or cKO mice and mounted on Grass Instruments FT03.C force transducers connected to a Powerlab 8/SP data acquisition unit (AD Instruments). A medium with physiological salt solution at 37°C and constant gas levels of 95% O_2_–5% CO_2_ was used for contraction studies. Electric stimulation with platinum electrodes was used to determine the optimal muscle length with maximal tetanic tension. After reaching optimal muscle length, muscles were stimulated for 20 seconds, and fatigue curves were generated. All measurements were normalized by muscle area, and specific force (mN/mm^2^) was used for analysis. For mechanical stretching experiments, the device was used to repeatedly stimulate EDL muscles for 30 minutes. Single myofibers were then isolated and immunostaining was performed.

### Histological analyses.

Skeletal muscle tissues were flash-frozen in tissue-freezing medium (Triangle BioSciences International) and gum tragacanth (MilliporeSigma, G1128) at a 3:1 ratio. The following histological stains were performed: routine H&E, NADH, COX, SDH, and metachromatic ATPase. SDH staining was performed with 0.2M phosphate buffer (pH 7.6) and sodium succinate with nitroblue tetrazolium chloride (NBT). NADH staining was performed with 0.05 M Tris buffer (pH 7.6) with NADH and NBT. COX staining was performed with 1 mg/ml cytochrome *c* (MilliporeSigma, C2506)/6 mg/ml catalase (MilliporeSigma, C40)/0.5 mg/ml 3,3-diamonobenzidine tetrachloride (MilliporeSigma, D5637) in PBS, pH 7.4. Metachromatic ATPase was performed on cryosections by sequential incubations in acid-differentiation medium (0.05M sodium acetate, 0.018M calcium chloride dihydrate, pH 4.4), TRIS buffer (0.1M Trizma base, 0.018M calcium chloride dihydrate, pH 7.8), ATP medium (0.05M glycine, 0.03M calcium chloride dihydrate, 0.07M sodium chloride, 0.05M sodium hydroxide, 0.004M adenosine 5′-trophosphate disodium salt hydrate, pH 9.4), 1% calcium chloride dihydrate, and 0.1% toluidine Blue (44). For trichrome staining, samples were fixed with 4% paraformaldehyde (Electron Microscopy Sciences, 15710) in PBS (MilliporeSigma, D8537) and embedded in paraffin. Cross sections were obtained and stained using Masson’s trichrome staining method. CD11b staining was performed followed a previously described protocol (45).

For immunofluorescence, frozen sections were fixed with 4% paraformaldehyde for 15 minutes, permeabilized with 0.3% Triton X-100 for 15 minutes, and blocked with MOM blocking solution (Vector Labs, BMK-2202) in 5% goat serum for 1 hour. Additional blocking was performed in 8% protein diluent (Vector Labs, BMK-2202) and 5% goat serum for 30 minutes. Primary antibodies were used at 1:200 dilution, and samples were incubated overnight at 4°C. Secondary antibodies were incubated with samples for 1 hour at room temperature.

The following antibodies were used: SUN2 (MilliporeSigma, MABT880), MYH7 (Developmental Studies Hybridoma Bank [DSHB], BA-D5), MYH2 (DSHB, SC-71), MYH4 (DSHB, BF-F3), MYH3 (DSHB, F1.652-s), γH2A.X (Cell Signaling Technology, 9718), MEF2C (Cell Signaling Technology, 5030), CD11B (Abcam, ab133357), DESMIN (Agilent, M076029-2), cleaved caspase-3 (Cell Signaling Technology, 9661) and WGA (Thermo Fisher, W11261), laminin (MilliporeSigma, L9393), and DAPI (MilliporeSigma, D9542). For TUNEL staining, Click-iT Plus EL Assay Kits for In Situ Apoptosis Detection (Thermo Fisher, C10617) were used per the manufacturer’s instructions. Imaging was performed using a Zeiss LSM 800 confocal microscope. ImageJ/Fiji (NIH) was used to measure myofiber diameter.

For electron microscopy, mice were perfused with 4% paraformaldehyde and 1% glutaraldehyde in 0.1M sodium cacodylate buffer (pH 7.4). Staining was done with 1% osmium tetroxide. Samples were processed by the UT Southwestern Center Electron Microscopy core facility. Images were acquired using a JEOL JEM-1400 Plus transmission electron microscope.

Generation and analysis of DesMef-LacZ reporter mice were previously described (31). X-gal staining of skeletal muscle was performed as previously described (46). Briefly, muscles were fixed in 2% paraformaldehyde/0.2% glutaraldehyde in PBS on ice for 1 hour and stained using 5 mM ferrocyanide, 5 mM ferricyanide, 2 mM MgCl_2_, and 1 mg/ml X-gal for 16 hours at room temperature. Washes were performed with PBS.

### Gene expression analysis.

For gene expression analysis in cells, samples were solubilized in 1 mL of TRIzol. RNA isolation was performed with the RNeasy Micro Kit (QIAGEN, 74004) per the manufacturer’s instructions. cDNA synthesis was performed with iScript Reverse Transcriptase (Bio-Rad, 1708841).

The following primers were used for qRT-PCR: mm.qPCR.Mef2c-F: GTCAGTTGGGAGCTTGCACTA; mm.qPCR.Mef2c-R: CGGTCTCTAGGAGGAGAAACA; mm.qPCR.Net39-F: ATCCTCTGCCTGGTGAGAA; and mm.qPCR.Net39-R: CCACTGTCATGATGTCCAAGAG. qRT-PCR primers for Atf3, Fas, Trp53, and Trp63 were ordered from MilliporeSigma (KiCqStart SYBR Green primers).

For bulk RNA-Seq, mRNA samples were sent to Genewiz for library preparation and paired-end 75 bp mRNA-Seq. For snRNA-Seq, sample preparation was performed with minor modifications from prior protocols (47). Nuclei from Ctrl and cKO GP muscles were isolated by mechanical dissociation. Nuclear preparations were loaded into the Chromium Next GEM Single Cell 3′ Gene Expression Kit, version 3.1 (10x Genomics) per the manufacturer’s instructions. Sequencing was performed at the Next Generation Sequencing Core of Children’s Research Institute at UT Southwestern on an Illumina NextSeq 500 system.

### ChIP-Seq.

Stable C2C12 myoblast cell lines (ATCC, CRL-1772) expressing TY1-NET39 or an empty vector were generated by retroviral infection. Five 15 cm plates of differentiated myotubes were used for each sample, and 2 replicates were used per biological group. Sample preparation was performed with the ChIP-IT High Sensitivity Kit (Active Motif, 53040) per the manufacturer’s instructions. Cells were fixed in cell-fixation solution for 30 minutes. A ChIP-grade Ty1 antibody (Diagenode, C15200054) was conjugated to protein G agarose beads for chromatin pulldown. Library preparation and sequencing were performed by the McDermott Center Next Generation Sequencing Core at the UT Southwestern Center.

### Bulk RNA-Seq analysis.

The FastQC tool (version 0.11.8) was used for quality control of RNA-Seq raw fastq data to evaluate low quality or adaptor portion of the reads for trimming. Reads were trimmed with Trimmomatic (version 0.39) and aligned to the mm10 reference genome using HiSAT2 (version 2.1.0) with default settings. The raw count matrix was generated using featureCounts (version 1.6.2) with bam files as input. DEG analysis between conditions was performed using the R package DESeq (version 1.38.0). Genes with fold change greater than 2 and adjusted *P* values of less than 0.05 were designated as DEGs between sample group comparisons. Enrichment analysis of gene sets was performed using the web resource Metascape (https://metascape.org).

### Net39-Ty1 ChIP-Seq analysis.

FastQC was used for quality control, and reads were aligned to the mm10 reference genome using bowtie2 (version 2.3.2) with default settings. Picard (version 2.10.3) was then used to remove duplicated reads. To increase sequencing depth for peak calling, replicates were merged for each condition. The LaminB1 DamID published data set was analyzed (6). To identify large domains of Net39- and/or LaminB1-associated regions, Enriched Domain Detector (EDD) (version 1.1.19) was used with the bin size set to 10 kb, and the output was the log ratio between ChIP and control for genome browser visualization. To calculate the correlation between Net39-Ty1 and lamin B1, a log ratio from EDD of 10 kb bins was used.

### Analysis of single-nucleus RNA-Seq data sets.

10× Cell Ranger (version 3.1.0) was used to perform sample demultiplexing and to generate the read count matrix with mm10/GRCm38 reference genome with default parameters. Further quality filtering was performed using the Seurat R package (version 3.2.2). Doublets were called using Scrublet (version 0.2.3) and removed from downstream analysis. Any barcodes (nuclei) with less than 100 gene features or with total read number less than 500 were filtered. Cells with more than 10% of the reads coming from mitochondrial genes were also filtered to remove potential dead cells from analysis.

Data were normalized using the SCTransform function from Seurat and vars.to.regress = “percent.mt” to remove potential mitochondrial effects. To integrate Ctrl and cKO data sets, 3,000 features were selected using the function SelectIntegrationFeatures, and the selected features were used as input for function PrepSCTIntegration. We then identified anchors and integrated the data set using the function FindIntegrationAnchor, with the normalization.method = “SCT” and anchor.features selected. Finally, the data sets were integrated using the function IntegrateData with the normalization.method = “SCT”.

For snRNA-Seq data visualization, dimensionality reduction was performed using PCA and uniform manifold approximation and projection (UMAP) with the functions RunPCA and RunUMAP, with dims set to 25. To cluster nuclei by their expression, graph-based clustering was done with the function FindNeighbors followed by FindClusters, with a resolution of 0.1, and 15 clusters were identified. After careful inspection with known cell markers, we annotated each cluster and merged clusters that did not show clear distinctions as the same cell type.

To generate heatmaps with bulk RNA-Seq and snRNA-Seq data side by side, DEGs from bulk RNA-Seq were intersected with genes that were detected in snRNA-Seq data. To correct dropout values in snRNA-Seq data, we used Markov affinity-based graph imputation of cells (MAGIC), which denoises the cell count matrix and fills in the missing values. After imputation, all heatmaps were combined to compare bulk RNA-Seq data with snRNA-Seq data.

### Western blotting.

Cell samples and flash-frozen tissues were processed with RIPA buffer (MilliporeSigma, R0278). The tissue lysates were further dissociated with mechanical dissociation (Precellys Evolution), and protein concentration was quantified by BCA assay (Thermo Fisher, 23225). Regular wet transfer was performed on polyvinylidene fluoride membranes (Millipore, IPVH00010). Blocking and antibody incubation were performed in 5% milk in TBS-Tween 0.1%. The following antibodies were used: GAPDH (MilliporeSigma, MAB374), α-tubulin (MilliporeSigma, T6199), NET39 (MilliporeSigma, HPA070252), MEF2C (Cell Signaling Technology, 5030), MYOD (Santa Cruz Biotechnology Inc., sc-377460), MYOG (Santa Cruz Biotechnology Inc., sc-12732 X), vinculin (VCL) (MilliporeSigma, V9131), HRP-conjugated streptavidin (Thermo Fisher, N100), γH2A.X (MilliporeSigma, 05-636), total ATM (Cell Signaling Technology, 2873T), and phosphorylated ATM (Ser1981) (Santa Cruz Biotechnology Inc., sc-47739). For blotting of total and phosphorylated ATM as well as γH2A.X, blocking was performed in 5% BSA in TBS-Tween 0.1%.

### In vitro studies.

C2C12 cells were cultured in growth media (DMEM with 10% fetal bovine serum and 1% antibiotic-antimycotic; Thermo Fisher, 26050088). Myoblast differentiation was induced with differentiation media (DMEM with 2% horse serum and 1% antibiotic-antimycotic). Selection of infected cells was performed in growth media with 2 μg/ml of puromycin (Thermo Fisher, A1113803). Paclitaxel (Thermo Fisher, P3456) was diluted in DMSO and used for microtubule stabilization at 10 nM and 50 nM.

Platinum-E ecotropic cells (Cell Biolabs, RV-101) and Lenti-X 293T cells (Takara, 632180) were used for the generation of retroviruses and lentiviruses, respectively. Cells were transfected with FuGENE6 (Promega, E2692) per the manufacturer’s instructions. pMD2.G (Addgene, 12259) and psPax2 (Addgene, 12260) were used for lentiviral packaging. Virus-containing media was collected 48 hours after transfection, filtered (0.45 μm membrane), and concentrated with Retro-X (Takara, 631456) or Lenti-X (Takara, 631232). Viral particles were resuspended in fresh media supplemented with 8 μg/ml of polybrene (MilliporeSigma, TR-1003-G) and used to infect C2C12 mouse myoblasts (ATCC, CRL-1772).

For knockdown studies, glycerol stocks of lentiviral plasmids (pLKO) containing shRNAs targeting Net39 were purchased from MilliporeSigma (TRCN0000081368, TRCN0000081369, TRCN0000081370, TRCN0000081371, TRCN0000081372). Scrambled shRNA cloned in pLKO was obtained from Addgene (no. 1864).

For proximity biotinylation (BioID), a custom retroviral backbone encoding the fusion protein NET39-miniTurbo from prior studies was used (48). C2C12 myoblasts expressing NET39-miniTurbo were differentiated into myotubes for 5 days in differentiation media. Myotubes were then exposed to 500 μM biotin (MilliporeSigma, B4501) for 4 hours to induce proximity biotinylation. Ctrl cells were treated with DMSO. Cells were lysed in 6M urea, 150 mM NaCl, and 10% SDS and lysed by homogenization (Bertin Instruments, P000062-PEVO0-A). Samples were diluted in 50 mM Tris, 150 mM NaCl, and incubated with 100 μl of streptavidin magnetic beads (Thermo Fisher, 88816) for 24 hours at 4°C. Beads were washed in lysis buffer and eluted in 2× Laemmli sample buffer (Bio-Rad, 1610737).

For luciferase assays, a luciferase reporter of Mef2 activity was used (49). The luciferase gene is under control of the *Des* enhancer containing 3 Mef2-binding sites. pMXs-puro-Net39-3xFLAG-HA (5) and pcDNA-Mef2c (50) were generated in previous studies. HEK293T cells were transfected with combinations of the reporter, Net39, and Mef2c plasmids. Samples were transfected with pCMV-LacZ for signal normalization. Luciferase assay system (Promega, E1500) and the Mammalian Beta Galactosidase Assay Kit (Thermo Fisher, 75707) were used for reporter measurements. Luminescence and absorbance readings were performed using a CLARIOstar plate reader (BMG Labtech).

A 6-well static cell confiner (4Dcell, CSOW 620) was used to apply mechanical stretch to C2C12 myoblasts. The device employs glass micropillars connected to PDMS pistons to compress cells in the vertical axis, thus generating horizontal mechanical stretch (16, 17). Cells were seeded onto glass-bottom 6-well plates (MatTek Corp., P06G-1.5-20-F). Prior to stretch, the PDMS pistons attached to micropillars were incubated in media for 1 hour, and then the cells were either unstretched or stretched for 1 hour. Following cell stretching, myoblasts were either treated with TRIzol for RNA isolation or fixed and processed for immunofluorescent staining. For rescue experiments, Mef2c and mcherry–DN-KASH were subcloned into a custom retroviral backbone. DN-KASH was amplified from pmcherry N1 (Addgene, 125553).

### AAV delivery of Net39.

AAV9 vectors expressing GFP-2A-Net39 or GFP-2A-TdTomato under control of the CK8e promoter were generated by conventional cloning from AAV9-CK8e-Cas9 (37). AAV packaging was performed at the Harvard Medical School/Boston Children’s Hospital Viral Core (Boston, Massachusetts, USA). AAV was delivered i.v. via facial vein at P2 to *Lmna* ΔK32 or Ctrl littermates and i.p. at P6 to *Net39*-KO mice at a concentration of 1 × 10^14^ vg/kg. *Lmna* ΔK32 mice were provided by Gisèle Bonne (Institut de Myologie, Paris, France) (7).

### Human EDMD analysis.

Three patients with *LMNA* mutations and available frozen muscle samples were selected and used both for this study and our prior study on *Net39* (5). The *LMNA* mutations were as follows: LMNA1, c.1357C>T, p.Arg453Trp, heterozygous; LMNA2, c.116G>A, p.Asn39Ser, heterozygous; and LMNA3, c.997_1110del, p.Thr333_Asp370del, heterozygous. Three additional age-matched samples from patients with no obvious muscle abnormalities were used as Ctrls. Frozen sections were cut from muscle biopsies and used for immunofluorescence studies.

### Data availability.

All high-throughput data in the manuscript were deposited in the NCBI’s Gene Expression Omnibus database (GEO GSE232049). Values for all data points found in graphs can be found in the supporting data values file.

### Statistics.

Statistical comparisons between 2 groups were evaluated by unpaired, 2-tailed Student’s *t* tests. A *P* value of less than 0.05 was considered statistically significant. Comparisons among 3 or more groups were evaluated by 1-way ANOVA followed by Tukey’s multiple-comparison analysis to determine the statistical significance among these groups. Two-way ANOVA was used to determine the statistical significance among 3 or more groups considering multiple factors. This analysis was followed by Tukey’s multiple-comparison analysis to determine the statistical significance among these groups. The log-rank (Mantel-Cox) test was performed to determine statistical significance among survival curves.

### Study approval.

The use of medical records and human tissues for research purposes was approved by the UT Southwestern Human Research Protection Program (IRB STU012016-082). This research was compliant with the ethical principles in the Belmont Report, Department of Health and Human Service human subject regulations, Title 21 CFR, and good clinical practice as adopted by the Food and Drug Administration. A waiver of patient informed consent was requested and approved by the human research protection program for retrospective study on archived human muscle tissue. All tissues used in this study were coded and deidentified. All animal procedures were approved by the Institutional Animal Care and Use Committee at the UT Southwestern Medical Center.

## Author contributions

ARM, YZ, NL, RBD, KC, LX, CC, and ENO wrote and edited the manuscript. CC supervised clinical research and provided patient samples. ARM, YZ, KC, and ENO designed the experiments and analyzed the data. ARM, YZ, MZD, FC, and ZW performed the experiments. JRM performed the zygote injections to generate Net39-cKO mice. ARM and YZ both contributed significantly and share co–first authorship. Authorship order was assigned based on the degree of contribution.

## Supplementary Material

Supplemental data

Supporting data values

## Version 1 07/03/2023

Electronic publication
