# Supplementary material for: Net39 protects muscle nuclei from mechanical stress during the pathogenesis of Emery-Dreifuss muscular dystrophy
Source: J Clin Invest. 2023 Jul 3;133(13):e163333. doi: 10.1172/JCI163333 (PMC10313361; doi:10.1172/JCI163333)
Supplement: Supplemental data [file jci-133-163333-s056.pdf]

Supplemental Figures

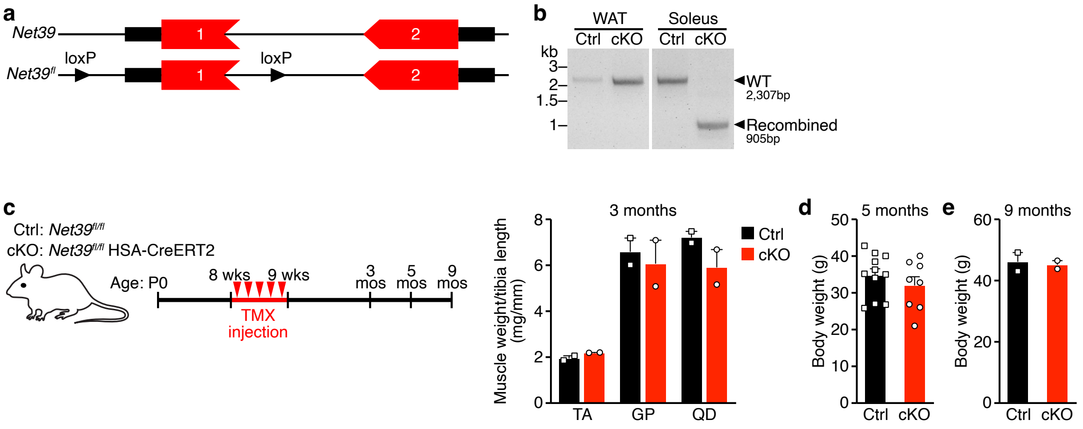

Supplemental Figure 1 – Inducible deletion of Net39 in adult skeletal muscle leads to progressive muscle wasting but no loss in body weight.

a) Gene structure of the conditional allele for *Net39* (*Net39* ^{fl}). Black arrowheads indicate the location of the loxP sequences inserted. Boxes represent exons. Black boxes denote untranslated regions and red boxes denote the open reading frame.

b) PCR analysis of recombination at the Net39 locus in Ctrl and cKO soleus and WAT. Wildtype (WT) allele is 2,307 bp and the recombined allele is 905 bp. WAT: White adipose tissue.

c) Experimental design for deletion of Net39 in adult skeletal muscle and analysis of these mice at 3 months, 5 months, or 9 months of age (left). Quantification of muscle weight for Ctrl and cKO mice at 3 months of age (right). TA: tibialis anterior, QD: quadriceps, GP: gastrocnemius-plantaris. Wks: Weeks, Mos: Months.

d) Quantification of body mass for Ctrl and cKO mice at 5 months of age.

e) Quantification of body mass for Ctrl and cKO mice at 9 months of age.

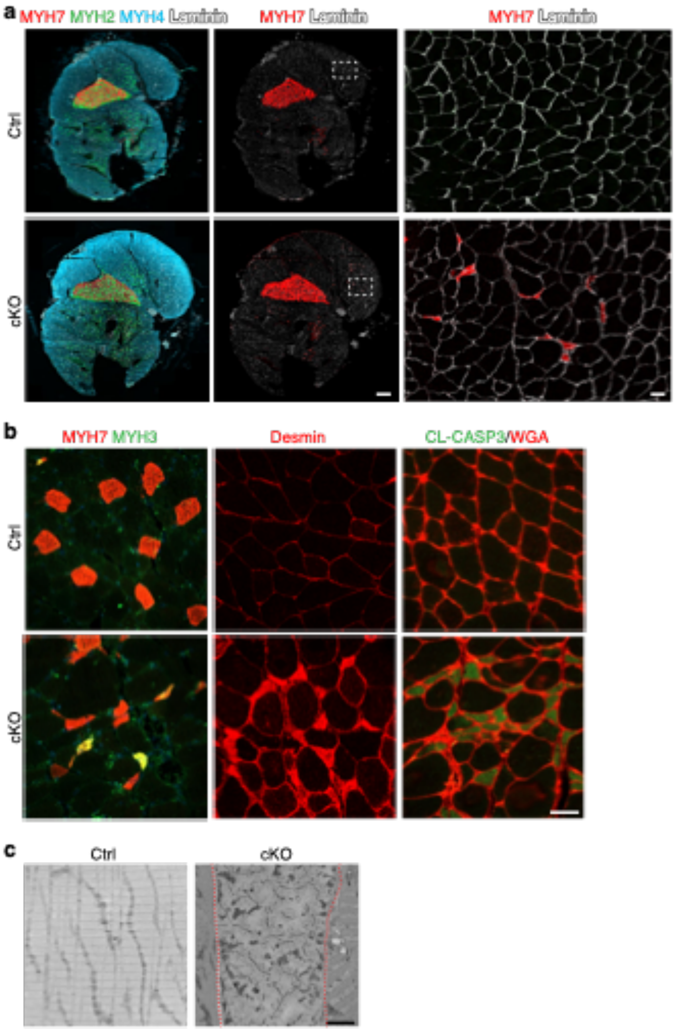

Supplemental Figure 2 –cKO muscle displays myopathic features.

a) Whole hindlimb immunostaining for type I (MYH7), type IIa (MYH2) and type IIb (MYH4) myofibers of Ctrl and cKO mice at 4 months of age. The magnified area (right) shows the presence of small angular fibers positive for MYH7 in cKO GP. Scale bar: left 500μm. Scale bar: right 50μm.

b) Immunostaining for type I (MYH7) and embryonic (MYH3) myosins (left), Desmin (middle), and cleaved caspase-3 (CL-CASP3) with WGA (right) in Ctrl and cKO mice at 5 months of age. Scale bar: 50μm**.**

c) Electron micrographs showing disorganized sarcomeres in cKO but not Ctrl GP muscle at 5 months of age. The small angular fiber with disorganized sarcomeres is outlined in the red borders. Scale bar: 4μm

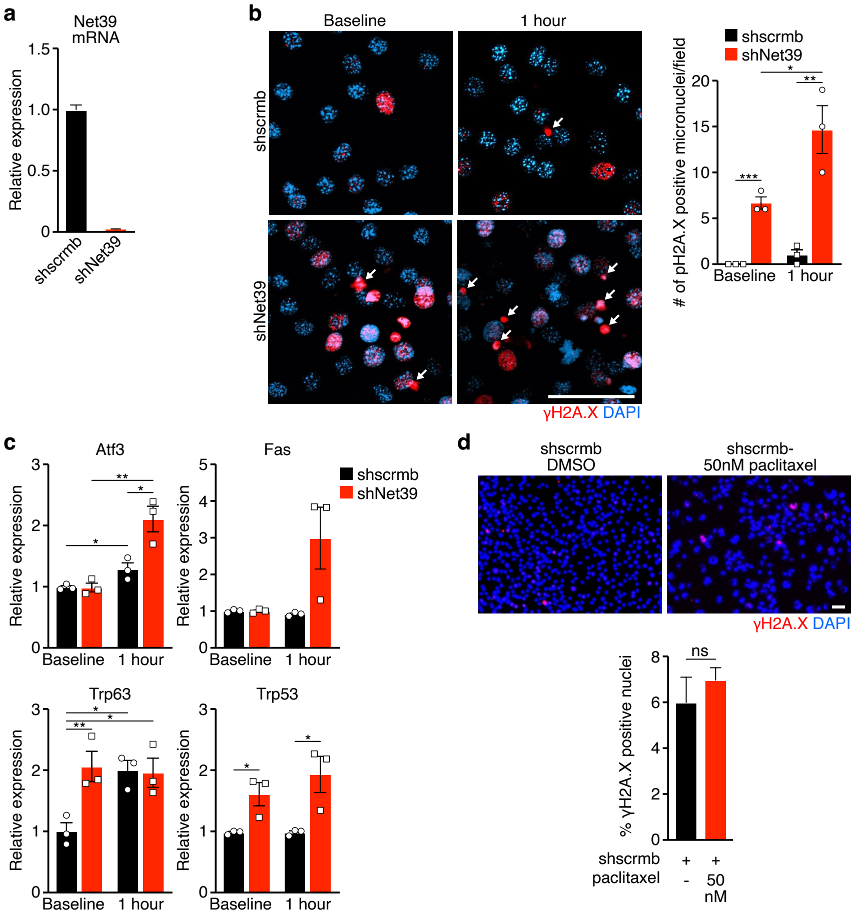

Supplemental Figure 3 – DNA damage is induced in shNet39 cells following compression.

a) mRNA expression of *Net39* in C2C12 myotubes expressing scrambled shRNA (shscrmb), as well as one shRNA targeting Net39 (shNet39).

b) γH2A.X-positive micronuclei are prevalent in shNet39 C2C12 myoblasts at baseline and after 1 hour of confinement. Quantification of the number of γH2A.X-positive micronuclei per field (right). White arrows indicate γH2A.X-positive micronuclei. * $p < 0.05$. ** $p < 0.01$. *** $p < 0.001$. Scale bar: 50μm.

c) mRNA expression of DNA damage-induced genes in shscrmb and shNet39 myoblasts at baseline and after 1 hour of confinement. Data were normalized to shscrmb cells at baseline. * $p < 0.05$. ** $p < 0.01$. Statistical comparisons between groups were evaluated by unpaired Student's t-test.

d) Immunostaining of γH2A.X in shscrmb C2C12 myoblasts following treatment with DMSO and 50nM paclitaxel. Quantification of percentage of γH2A.X-positive nuclei (bottom). Statistical comparisons between groups were evaluated by unpaired Student's t-test. Ns – Not significant. Scale bar: 50μm.

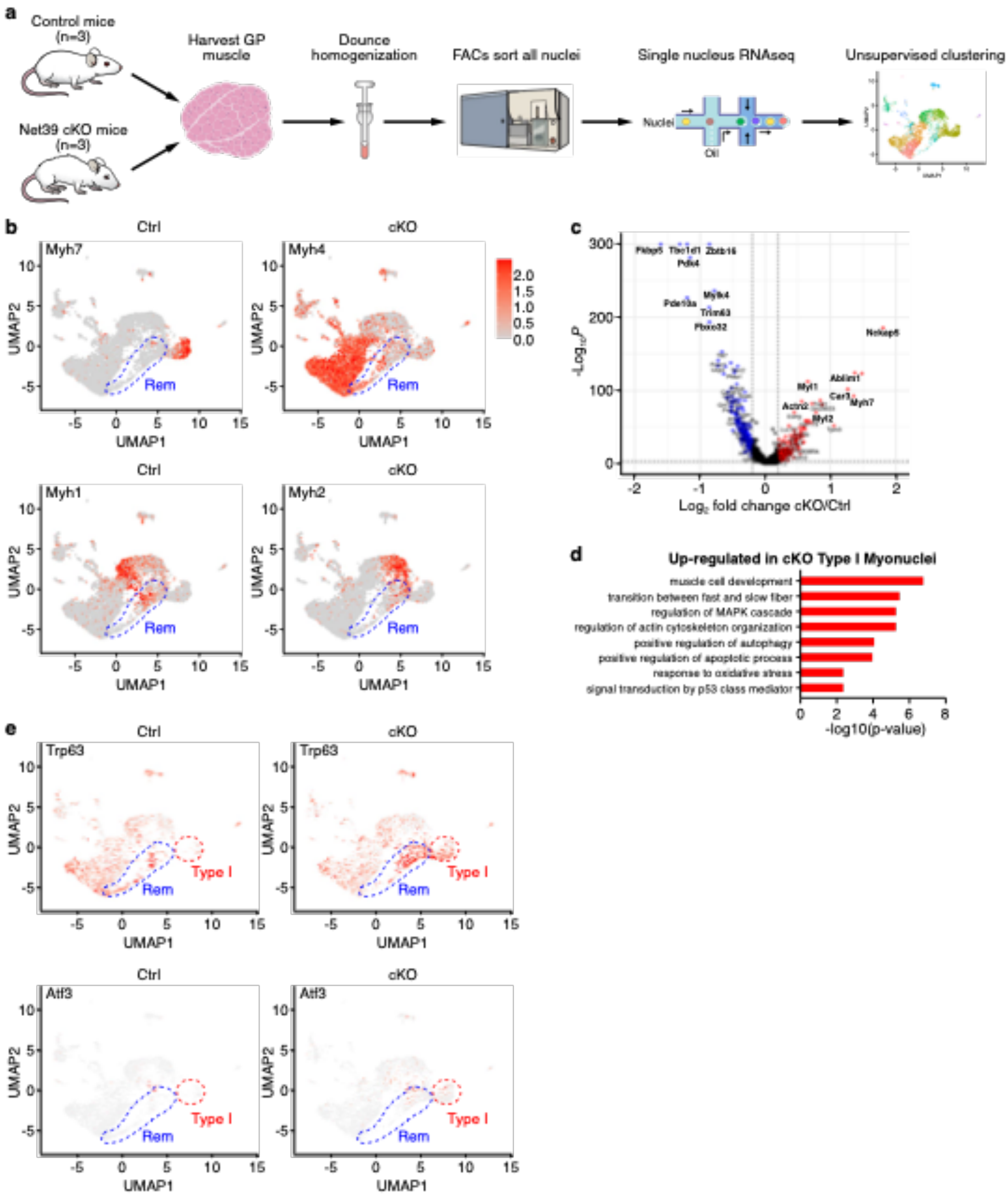

Supplemental Figure 4 – single nucleus RNA-seq identifies that pathological myonuclei express higher levels of DNA damage-induced genes.

a) Schematic of the experimental design for snRNA-seq on skeletal muscle nuclei from Ctrl and cKO mice.

b) UMAP visualization of Ctrl and cKO expression of different myosin isoforms from snRNA-seq data. Remodeling myonuclei show expression of *Myh7*, *Myh1, Myh2, and Myh4*. Remodeling myonuclei are enclosed in blue.

c) Volcano plot illustrating the up- and down-regulated genes in Ctrl and cKO GP myonuclei at 5 months of age by snRNA-seq.

d) Pathway analysis of the upregulated genes in cKO Type I myonuclei at 5 months of age by snRNA-seq.

e) UMAP visualization of Ctrl and cKO expression of the DNA damage-related genes *Atf3* and *Trp63*, which showed an enrichment in cKO remodeling and type I myonuclei. Remodeling myonuclei are enclosed in blue. Type I myonuclei are enclosed in red.

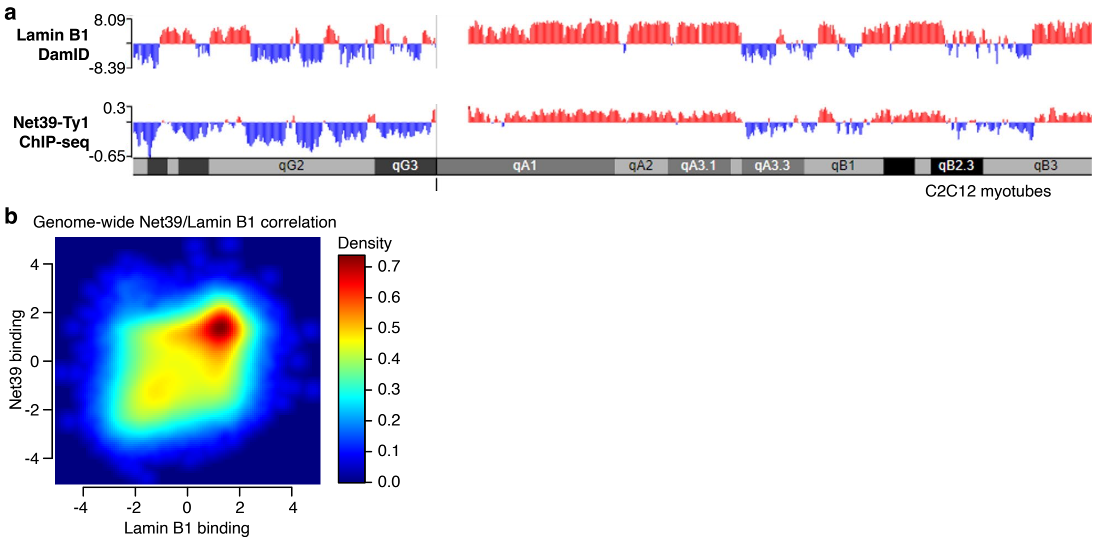

Supplemental Figure 5 – Net39 binds DNA regions that resemble Lamin-associated domains

a) LaminB1 DamID and Net39-Ty1 ChIPseq tracks shown for chromosome 6. Red indicates called Lamin B1 and Net39-Ty1 peaks. Chromosome arms are shown at the bottom.

b) Correlation of Lamin B1 and Net39-Ty1 peaks for all chromosomes. X- and Y-axis values represent log ratios of ChIP signal to control for Net39-Ty1 and Lamin B1, respectively.

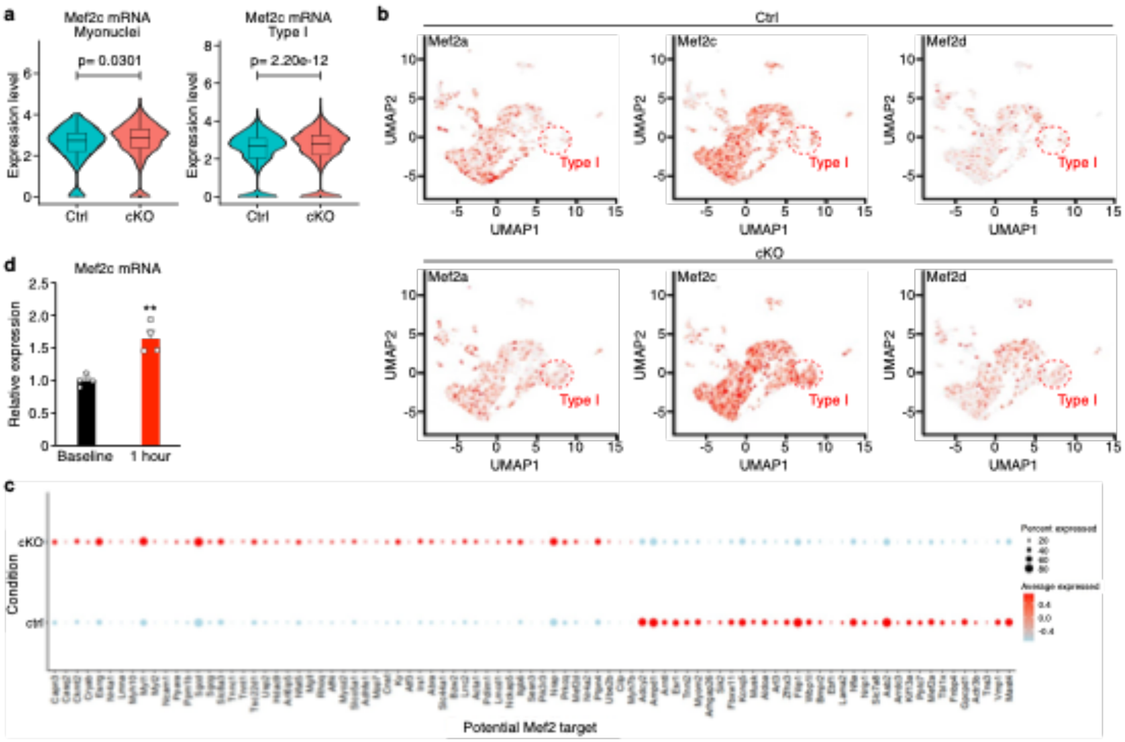

Supplemental Figure 6 – Mef2c is induced in cKO myonuclei along with its target genes.

a) Violin plot showing the expression of *Mef2c* in myonuclei (left) and Type I myonuclei (right). P-values for expression comparison between Ctrl and cKO are shown.

b) UMAP visualization of Ctrl and cKO expression of *Mef2* family members *Mef2a*, *Mef2c*, and *Mef2d* expression. Type I myonuclei are enclosed in red.

c) Dot-plots from snRNA-seq showing the expression of Mef2c target genes differentially expressed in Ctrl vs. cKO myonuclei. Red dots indicate upregulated genes and blue dots indicate downregulated genes in cKO.

d) *Mef2c* mRNA expression in shscrmb C2C12 myoblasts at baseline and after 1 hour of confinement. **$p < 0.01$. Statistical comparisons between groups were evaluated by unpaired Student's t-test.

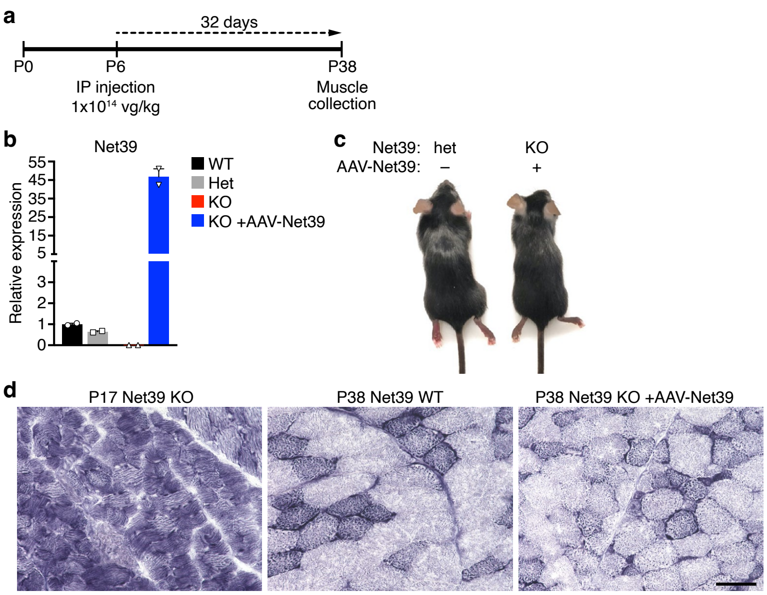

Supplemental Figure 7 – AAV-Net39 injection rescues lethality and failure to thrive in Net39 KO mice.

a) Experimental plan for delivery of AAV-Net39 into Net39 global knockout (KO) mice. $1x10^{14}$ viral genome (vg)/kilogram mouse weight (kg) of AAV was injected intraperitoneally (IP) at P6 and muscles were collected at P38.

b) *Net39* mRNA expression in GP muscles from Net39 KO mice at P38 injected with AAV-Net39 compared with wildtype (WT), heterozygote (Het), and KO mice.

c) Images of Net39 Het and KO mice uninjected (-) and injected (+) with AAV-Net39.

d) NADH staining of GP muscles of P17 Net39 KO mice, P38 Net39 WT mice, and P38 Net39 KO mice injected with AAV-Net39. Scale bar: 50μm.

a

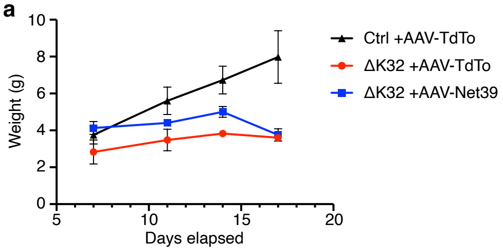

Supplemental Figure 8 – AAV-Net39 injection improves ΔK32 body weight.

a) Growth curves showing the body weight of Ctrl (black) and ΔK32 mice injected with AAV-TdTo (red) or AAV-Net39 (blue).
